# Co-occurrence of *Campylobacter* Species in Children From Eastern Ethiopia, and Their Association With Environmental Enteric Dysfunction, Diarrhea, and Host Microbiome

**DOI:** 10.3389/fpubh.2020.00099

**Published:** 2020-04-15

**Authors:** Yitagele Terefe, Loïc Deblais, Mostafa Ghanem, Yosra A. Helmy, Bahar Mummed, Dehao Chen, Nitya Singh, Vida Ahyong, Katrina Kalantar, Getnet Yimer, Jemal Yousuf Hassen, Abdulmuen Mohammed, Sarah L. McKune, Mark J. Manary, Maria Isabel Ordiz, Wondwossen Gebreyes, Arie H. Havelaar, Gireesh Rajashekara

**Affiliations:** ^1^The Ohio State University, Columbus, OH, United States; ^2^Veterinary Medicine, Haramaya University, Dire Dawa, Ethiopia; ^3^Global One Health Initiative, The Ohio State University, Addis Ababa, Ethiopia; ^4^Department of Environmental and Global Health, University of Florida, Gainesville, FL, United States; ^5^Emerging Pathogens Institute, University of Florida, Gainesville, FL, United States; ^6^Chan Zuckerberg Biohub, San Francisco, CA, United States; ^7^Chan Zuckerberg Initiative, Redwood City, CA, United States; ^8^Department of Rural Development and Agricultural Extension, Haramaya University, Dire Dawa, Ethiopia; ^9^Department of Pediatrics, Washington University, St. Louis, MI, United States

**Keywords:** *Campylobacter*, non-thermotolerant *Campylobacter*, EED, diarrhea, malnutrition, stunting, livestock reservoirs, MeTRS

## Abstract

High *Campylobacter* prevalence during early childhood has been associated with stunting and environmental enteric dysfunction (EED), especially in low resource settings. This study assessed the prevalence, diversity, abundance, and co-occurrence of *Campylobacter* spp. in stools from children in a rural area of eastern Ethiopia and their association with microbiome, diarrhea, and EED in children. Stool samples (*n* = 100) were collected from randomly selected children (age range: 360–498 days) in five kebeles in Haramaya District, Ethiopia. Diarrhea, compromised gut permeability, and gut inflammation were observed in 48, 45, and 57% of children, respectively. *Campylobacter* prevalence and species diversity were assessed using PCR and meta-total RNA sequencing (MeTRS). The prevalence of *Campylobacter* spp. in the children's stools was 50% (41–60%) by PCR and 88% (80–93.6%) by MeTRS (*P* < 0.01). Further, seven *Campylobacter* species (*Campylobacter jejuni, Campylobacter upsaliensis, Campylobacter hyointestinalis, Campylobacter coli, Campylobacter* sp. RM6137, uncultured *Campylobacter* sp., and *Campylobacter* sp. RM12175) were detected by MeTRS in at least 40% of children stools in high abundance (>1.76-log read per million per positive stool sample). Four clusters of *Campylobacter* species (5–12 species per cluster) co-occurred in the stool samples, suggesting that *Campylobacter* colonization of children may have occurred through multiple reservoirs or from a reservoir in which several *Campylobacter* species may co-inhabit. No associations between *Campylobacter* spp., EED, and diarrhea were detected in this cross-sectional study; however, characteristic microbiome profiles were identified based on the prevalence of *Campylobacter* spp., EED severity, and diarrhea. Forty-seven bacterial species were correlated with *Campylobacter*, and 13 of them also correlated with gut permeability, gut inflammation and/or EED severity. Forty-nine species not correlated with *Campylobacter* were correlated with gut permeability, gut inflammation, EED severity and/or diarrhea. This study demonstrated that (1) in addition to *C. jejuni* and *C. coli*, multiple non-thermophilic *Campylobacter* spp. (i.e., *Campylobacter hyointestinalis, Campylobacter fetus*, and *Campylobacter concisus*) were frequently detected in the children's stools and (2) the *Campylobacter*, gut permeability, gut inflammation, EED severity, and diarrhea were associated with characteristic microbiome composition. Additional spatial and longitudinal studies are needed to identify environmental reservoirs and sources of infection of children with disparate *Campylobacter* species and to better define their associations with EED in low-income countries.

## Introduction

*Campylobacter* species are the most common zoonotic pathogens and the most frequent bacterial cause of foodborne disease worldwide (1). *Campylobacter* infection is frequently asymptomatic, and clinical cases may present with symptoms ranging from diarrhea, abdominal pain, and fever to severe consequences like reactive arthritis and, although rarely occurring, Guillain–Barré syndrome (2). Among the cases of diarrhea in children <5 years old, 15% was caused by *Campylobacter* infections (3). Warm-blooded animals, and particularly avian species, are common reservoir hosts for *Campylobacter* and infection in animals in most cases is asymptomatic. *Campylobacter* transmission from animal reservoirs to humans may occur through multiple routes, including contaminated food (especially poultry meat) and water, the environment, and contact with infected animals (4, 5). Children can be exposed to *Campylobacter* spp. directly or indirectly through exposure to animal feces (6).

Recent studies have also shown association of both symptomatic and asymptomatic *Campylobacter* infections with growth faltering in children from developing countries (7, 8). Approximately 24 million (35.2%) children under five, from East Africa were stunted, 4.1 million (6%) were wasted, and 2.9 million (4.3%) were overweight in 2018 (9). High exposure to enteric pathogens may result in environmental enteric dysfunction (EED), a subclinical disorder of the small intestine characterized by villous atrophy, crypt elongation, inflammatory cells infiltration of the crypts and a loss of barrier function or increased permeability (10). EED is considered to be involved in the causal pathway from pathogen exposure to stunting (11, 12). A study in multi-country settings, *Campylobacter* spp. were isolated in both diarrheic and non-diarrheic children in their first and second year of life (13). In addition to these, the MAL-ED project revealed a high *Campylobacter* infection in children in eight low-resource settings and this was associated with growth shortfalls, increased intestinal permeability, and intestinal and systemic inflammation at 24 months of age (7).

In Ethiopia, the rate of stunting among children under five is alarmingly high (38% in 2016); and a recent study indicated that the average height-for-age Z-score (HAZ) in a large sample of Ethiopian children decreased from −0.7 to −2.0 standard deviations between 6 and 18 months of age (14). In previous studies, high prevalence of thermophilic *Campylobacter* spp. in humans and domestic animals have been documented (15–18). Studies from Gondar, Hawassa and Jimma, Ethiopia indicated that *Campylobacter* was a major cause of diarrhea in children <5 years (15, 16) with high prevalence of *Campylobacter* in children who had exposure to domestic animals mainly chicken (15). Evidence are still lacking in Ethiopia concerning the association between *Campylobacter* spp. and EED in children. Thermotolerant *Campylobacter* spp. (i.e., *Campylobacter jejuni, Campylobacter coli, Campylobacter lari*, and *Campylobacter upsaliensis*) are frequent causal agents of campylobacterioisis. However, infection of children with non-thermophilic *Campylobacter* spp. (e.g., *Campylobacter hyointestinalis, Campylobacter fetus, Campylobacter showae*, and *Campylobacter concisus*) is largely underestimated due to unsuitable culturing methods and their public health risks specifically in EED pathogenesis is not known (19, 20).

Improving gut health in children can improve growth and cognitive development and the efficacy of oral vaccines (21). The fight against stunting and wasting is the prioritized agenda of several regional, national and international interventions in low and middle-income countries (22). Understanding the causes of EED and specifically characterizing the associated pathogens provide opportunities to design effective interventions to improve the health and well-being of children. Therefore, the current study is aimed at estimating the prevalence, abundance, diversity, and co-occurrence of *Campylobacter* spp. in children stools collected in the Haramaya District/Woreda, East Hararghe zone, Oromia region, in rural eastern Ethiopia, and to assess their association with EED, diarrhea, and host microbiome. This study is a part of the cross-sectional formative research of the *Campylobacter* Genomics and Environmental Enteric Dysfunction (CAGED) project, which included an epidemiological investigation of *Campylobacter* exposure, EED, stunting, and their associated risk factors. Results provided by this cross-sectional study will provide a strong baseline for examining the role of *Campylobacter* in EED.

## Materials and Methods

### Study Area

**Figure 1:**
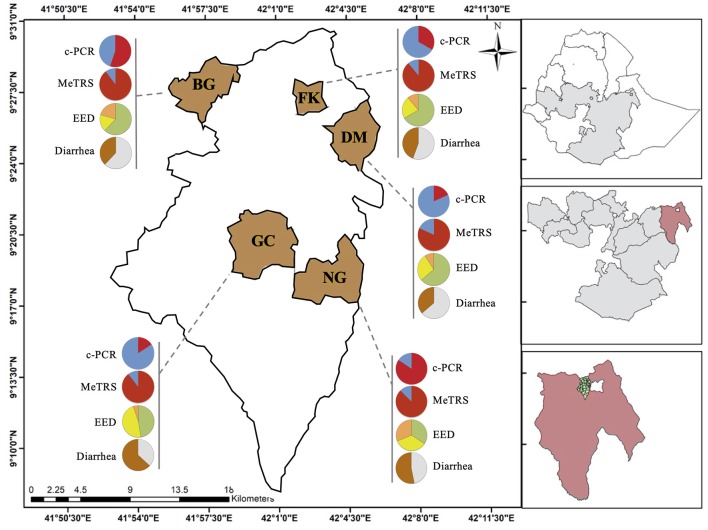
Prevalence of *Campylobacter*, environmental enteric dysfunction (EED) and diarrhea in children in the five kebeles from Haramaya District (East Ethiopia). Pie graphs in red (positive for *Campylobacter*) and blue (negative for *Campylobacter*) represent the *Campylobacter* prevalence in stool samples (*n* = 100) collected from the designated kebele. Prevalence was determined using conventional PCR (c-PCR) or MeTRS (meta-total RNA sequencing). Pie graphs in green (“normal”), yellow (“moderate” EED), and orange (“severe” EED) represent the prevalence and severity of the EED for the designated kebele. Additional details concerning the EED severity determination are presented in Table S1. Pie graphs in brown (child currently having or had diarrhea in the past 15 days), gray (child had no diarrhea in the past 15 days) represent the diarrheal status of the children. GC, Gobe Chala; NG, Negaya; FK, Finkle; DM, Damota; BG: Biftu Geda.

The study was conducted in five rural kebeles (Biftu Geda, Damota, Finkile, Gobe Chala, and Negeya; smallest administrative unit in Ethiopia) in the Haramaya District/Woreda, East Hararghe zone of Oromia Regional state, Eastern Ethiopia (Figure 1). Haramaya District is located about 525 km from Addis Ababa (capital city of Ethiopia). The altitude of Haramaya District ranges from 1,400 to 2,340 meters above sea level. Haramaya District has 36 rural kebeles and three urban kebeles. The national census of 2007 reported a total population of 271,018 (138,282 men and 132,736 women) (23) for this District. A survey of the land in Haramaya District shows that 36.1% is arable or cultivable, 2.3% pasture, 1.5% forest, and the remaining 60.1% is considered built-up, degraded, or otherwise unusable. The livestock population of the Haramaya District was estimated as; 111,528 cattle, 69,950 sheep, 106,145 goats, 137,545 chickens, 529 camels, and 31,385 donkeys. Khat, vegetables, and fruits represent major cash crops of this district (Haramaya District Agriculture and Livestock office; unpublished data).

### Study Design and Sample Size Calculation

A cross-sectional study was conducted on children between October 2018 to December 2018 (age range: 360–498 days). For the prevalence estimation, sample size was calculated based on a binomial distribution. A sample of 100 children allows estimation of 50% prevalence with a precision of 10% at 95% confidence interval, and a power of 80%. The target number of 100 children was distributed over the five kebeles (between 9 and 32 children per kebeles) based on the number of children in the sampling frame but rounded to the nearest multiple of 10. Full details of the enrollment process are described in Chen et al. (under review).

### Sample Collection and Transportation

Stool samples were collected weekly from 10 children in one of the selected kebeles over 3 month period until the desired sample size (*n* = 100) was achieved. Child caretakers were invited to bring the children to the local health post for stool sampling. The samples were collected in a clean plastic sheet and then immediately transferred into four FluidX™ 2D-Barcoded 2.0 mL sample storage tubes (Thermo Scientific™, Waltham, MA, USA) and kept in an ice box and transferred to the laboratory at Haramaya University on the same day. Sample ID, date, and time of collection were verified, and samples were stored at −80°C until further use for DNA extraction. The child caretaker was asked whether the child had diarrhea during the 15 days before collection. Additional details concerning the diarrhea scoring are described in Chen et al. (under review).

### Prevalence and Measurement of EED

Prevalence of EED and its measurements are described in detail in Chen et al. (under review). EED was determined by assessing both gut permeability and inflammation. The lactulose absorption (L%) was measured via sugar absorption test, as previously described (24), and was used as a maker to assess the gut permeability of the children. Classification of EED severity was performed as previously described (25). The child was considered “normal” if the L% was lower than 0.2; “moderate” if the L% was between 0.2 and 0.45, and “severe” if the L% was above 0.45. Myeloperoxidase (MPO) in the stool samples was measured using a commercially available enzyme linked immunosorbent assay (MPO RUO, Alpco, Salem, NH) to assess the gut inflammation. The child was considered having “normal” gut integrity if the [MPO] was lower than 2,000 ng/ml, “moderate” gut inflammation if the [MPO] was in between 2,000 and 11,000 ng/ml, and “severe” gut inflammation if the [MPO] was above 11,000 ng/ml. Due to the lack of correlation between the abundance of *Campylobacter* in stool and lactulose absorption and MPO data (*r*^2^ < 0.2; *P* > 0.05), the EED severity in the children was estimated by cross-tabulating the classifications generated for both L% and [MPO] (Figure S1).

### Extraction of Genomic DNA

Extraction of the genomic DNA from stool samples was performed by using Purelink^TM^ Microbiome DNA purification kit (Invitrogen, Carlsbad, CA, USA). Traces of RNA were removed using RNAse treatment (Thermo Scientific™, Waltham, MA, USA) as previously described (26, 27). Genomic DNA was resuspended in 50 μl of nuclease free water (Qiagen, CA, USA) and stored at −20°C for further use. Quality and quantity of the extracted DNA was assessed using 1.5% agarose gel electrophoresis and Nanodrop 2000C Spectrophotometer (Thermo Scientific™, Waltham, MA, USA).

### Detection of *Campylobacter* spp. in child Stool Samples Using Conventional PCR

Detection of *Campylobacter* spp. in DNA from stool samples was performed using multiplex conventional PCR with the GoTaq Green Master Mix kit (Promega Life Sciences, Madison, WI, USA). *C. jejuni* 81–176, *C. coli* ATCC33559, and sterile water were used as controls. *Campylobacter* genus-specific PCR was performed using 16S RNA primers and *Campylobacter* species-specific PCR was performed using *ceuE* primers for the detection of *C. coli* and *mapA* primers for the detection of *C. jejuni* (28). The primers used and the expected PCR product sizes are described in Table S1. The PCR was performed as described in Denis et al. (28) in a Mastercycler nexus gradient PCR system (Eppendorf, Hamburg, Germany). PCR products were visualized using 1.5% agarose gel (VWR International, Radnor, PA, USA) under UV light.

### RNA Extraction, Library Preparation and Sequencing

RNA extraction was performed using approximately 0.25 g of stool sample with the Quick-RNA Fecal/Soil Microbe Microprep Kit (Zymo Research, CA, USA) according to the manufacturer's protocol. Four water samples were used as controls during extraction and library preparation. RNA concentration was measured using Qubit (Invitrogen, Carlsbad, CA, USA). Meta-total RNA sequencing (MeTRS) was used to analyze the microbiome composition of the children stools based on previously published work (29). The library generation was performed with NEBNext® Ultra™ II RNA Library Prep (New England Biolabs, MA, USA). Sequencing was performed to obtain ~400M reads using the Illumina NextSeq (Illumina, Inc., San Diego, CA, USA) across the 100 stool samples. For each sample, 25 pg of External RNA Controls Consortium (ERCC) RNA Spike-In Mix (Life Technologies, Carlsbad, CA, USA) was added prior to library preparation to determine the limit of detection for each sample. The average lower limit of detection was 49 attomoles. The average RNA input per samples was calculated using the following equation (total input RNA = ercc_pg/ercc_reads ^*^ total_reads) where “ercc_reads” is the number of reads generated from the water control and “total_reads” is the number of reads generated from test RNA including the water control. The average input was ~66 ng/sample.

### Bioinformatic Analysis of the MeTRS Data

MeTRS data analysis was performed using IDseq pipeline version 3.7 available at https://github.com/chanzuckerberg/idseq-web/wiki (30). The details of the IDseq pipeline used for MeTRS data analysis is included in the Supplemental Material 1. Only identified organisms with at least 10 reads, an alignment length above 50 bp, and a Z-score above 1 were considered for the statistical analyses.

### Statistical Analysis

MeTRS abundance data (reads per million; rpm) were log transformed. Statistical analyses were performed using JMP PRO 14 software (SAS Institute, Cary, NC, USA). The homogeneity of the MeTRS data for each child was analyzed using a principal component analysis (PCA) combined with restricted maximum likelihood (REML) estimation and *T*^2^ statistic test (square of the Mahalanobis distance). Similarity in the *Campylobacter* spp. profiles between children obtained with the MeTRS data was determined using hierarchical clustering. Discriminant analyses were performed to identify specific members of the microbial species responsible for the variability observed in the PCA and clustering data based on the designated nominal parameter used (kebeles, presence/absence of *Campylobacter*, EED severity, and diarrhea status). The percentage of stool samples clustering within their own group (confidence interval of 95%; CI 95%) was used to determine whether the designated nominal parameters explained the variability observed between stool samples. A Wilcoxon test was performed to identify rpm abundance differences for a given member of the microbial species based on a specific nominal parameter. Correlations between the MeTRS, EED severity, and diarrhea data were performed using a multivariate analysis combined with Pearson product-moment correlation coefficient. The co-occurrence of the *Campylobacter* spp. was studied using Cluster and Factoextra R packages (SAS Institute, Cary, NC, USA) on the K-means clustering data extracted from the multivariate analysis data (*r*^2^) obtained with the *Campylopbacter* spp. prevalence data. The optimization of the number of clusters for the PCA was performed using the Silhouette method. The abundance of each bacteria in the stool samples was estimated using MeTRS approach, which is based on RNA read quantification; therefore, we do not exclude the possibility that this approach over or under estimates the quantitative data (reads per million) described in this study.

### Data Accession

MeTRS raw reads have been deposited in the IDseq platform (https://idseq.net) in accordance with the Chan Zuckerberg Biohub and Chan Zuckerberg Initiative (CZI).

## Results

### Prevalence of *Campylobacter* spp. in child Stool Using Conventional PCR

The genus-specific PCR analysis showed that 50% (40–60%, 95% CI) of the child stools (*n* = 100) were positive for the *Campylobacter* genus across the five kebeles (Table 1); however, the *Campylobacter* prevalence significantly differed between kebeles (*P* < 0.05; Figure 1 and Figure S2A). The prevalence was higher in Negeya (84.4%) compared to Biftu Geda (55.2%), followed by Finkile (33.3%), Damota (18.2%) and Gobe Chala (15.8%; Figure 1 and Figure S2A).

**Table 1:** Prevalence of *Campylobacter, Campylobacter jejuni*, and *Campylobacter coli* in child stools using conventional PCR and MeTRS approaches.

**Taxonomic group**	**Conventional PCR**		MeTRS^*^	
	**Positive samples**	**Prevalence (%)**	**Positive samples**	**Prevalence (%)**
*Campylobacter* genus	51	50 (40-61)	88	88 (80–93.6)
*C. jejuni*	13	13 (7-21)	37	37 (27-46)
*C. coli*	2	2 (0.2–7)	24	24 (17-32)

* *samples were considered positive for Campylobacter spp. if at least 10 reads per sample of at least 50 bp long were mapped to reference genome with a Z-score higher than 1*.

The species-specific PCR analysis showed that 13% (7–21%) of children were colonized with *C. jejuni* and only 2% (0.2–7%) with *C. coli* (Table 1). The prevalence of children positive for *C. jejuni* and *C. coli* did not differ between kebeles (Figure S2A). These results suggested that large proportion of *Campylobacter* in children stools likely represent other *Campylobacter* species, possibly including non-thermotolerant *Campylobacter*.

### Prevalence of *Campylobacter* spp. in child Stools Using Meta-Total RNA Sequencing

**Figure 2:**
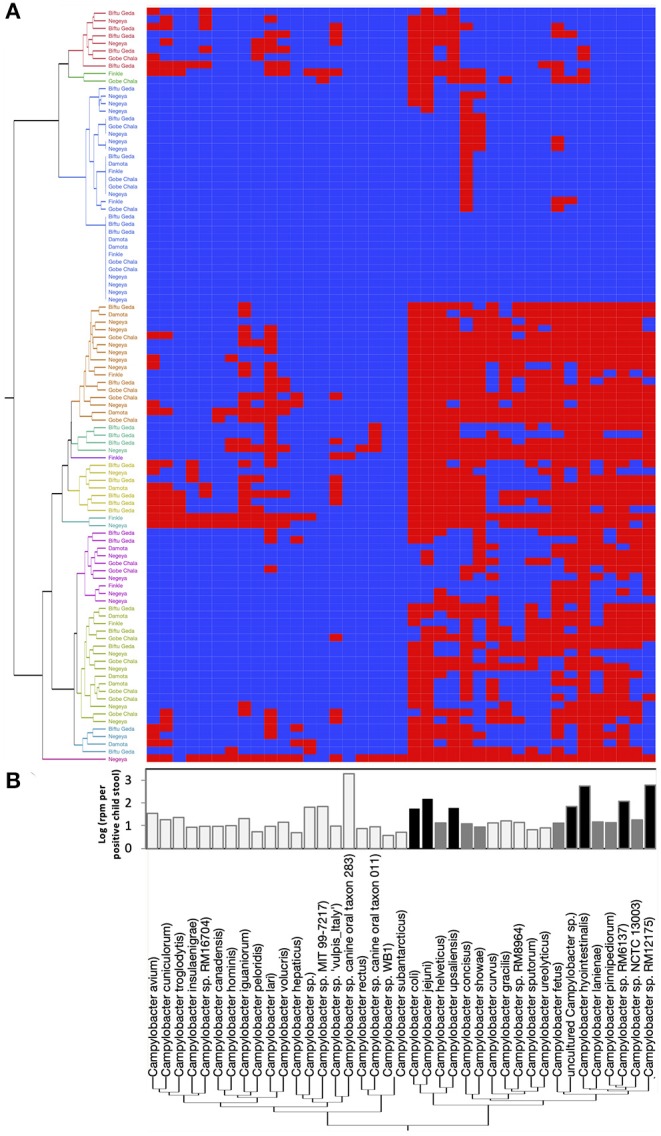
*Campylobacter* spp. prevalence, diversity, and abundance in children stools. A total of 27 classified *Campylobacter* spp. and 12 unclassified *Campylobacter* spp. were detected in the 100 child stool samples collected from children in the five kebeles using MeTRS. **(A)** *Campylobacter* spp. prevalence and diversity in the stool samples. Blue and red cells represent the absence or presence of *Campylobacter* spp. in the designated stool samples (cut-off; contigs number ≥ 10; Read length ≥ 50; Z-score ≥ 1). Kebeles with the same color code belong to the same cluster and therefore harbored equivalent *Campylobacter* sp. diversity. **(B)** Abundance of *Campylobacter* spp. in the positive stools. White bar represents *Campylobacter* spp. with a prevalence lower than 40% and an abundance lower than 0.95-log rpm per stool sample. Gray bar represents *Campylobacter* spp. with a prevalence higher than 40% and an abundance lower than 1.76-log rpm per stool sample. Black bar represents *Campylobacter* spp. with a prevalence higher than 40% and an abundance higher than 1.76-log rpm per stool sample. rpm: read per million.

In order to identify the full spectrum of *Campylobacter* species present in the stool samples, MeTRS was performed on 100 children stool samples. The MeTRS analysis showed that *Campylobacter* was detected in 88% (95% CI: 80–94%) of the children's stools (Table 1). A total of 27 classified *Campylobacter* species and 12 unclassified *Campylobacter* species were detected among the 88 *Campylobacter* positive stools based on the annotation in the NCBI database with average sequence identity of 97% for each species (https://www.ncbi.nlm.nih.gov/Taxonomy/Browser/wwwtax.cgi; Figure 2). An average of 11 *Campylobacter* spp. was detected per *Campylobacter* positive stool. Seven *Campylobacter* spp. (from highest to lowest prevalence; *Campylobacter* sp. RM12175, *C. hyointestinalis, C. jejuni, Campylobacter* sp. RM6137, uncultured *Campylobacter* sp*., C. upsaliensis*, and *C. coli*) were detected in at least 40% of the stools at high abundance (at least 1.76-log rpm per positive stool; Figure 2); and seven other *Campylobacter* spp. (*Campylobacter* sp. NCTC 13003*, Campylobacter helveticus, Campylobacter lanienae, C. concisus, C. fetus, Campylobacter pinnipediorum*, and *C. showae*) were detected in at least 40% of the stools but at lower abundance (between 0.95-log and 1.76-log rpm per positive stool; Figure 2).

**Figure 3:**
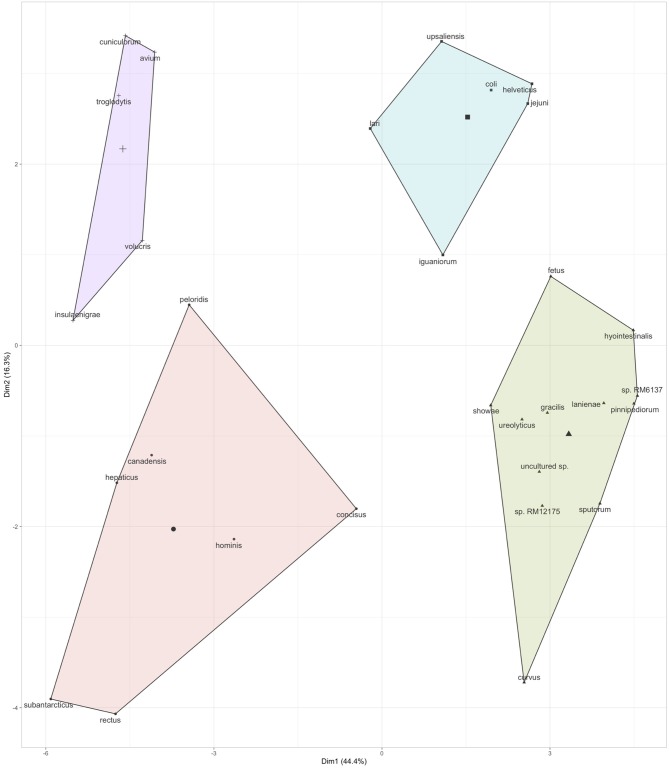
Co-occurrence of *Campylobacter* spp. in the children stool samples. Co-occurrence profiles were created using K-means clustering data extracted from the multivariate analysis data (*r*^2^) based on the prevalence of *Campylobacter* spp. in the stool samples (see Figure 2). Additional details regarding the multivariate data are displayed in Figure S3.

Further, based on the prevalence data (Figure 2), it was also observed that specific *Campylobacter* spp. often co-occurred in the children stools (Figure 3 and Figure S3). A total of four clusters of co-occurrences (*n* = 5–12 *Campylobacter* spp. per cluster) were detected. The green cluster (*C. coli, C. jejuni, C. helveticus, C. upsaliensis C. iguaniorum*, and *C. lari)* and the red cluster (*C. gracillis, C*. sp. RM12175, *C. sputorum*, and *C. ureolyticus, C. hyointestinalis, C. curvus, C. fetus, C. lanienae, C. pinnipediorum, Campylobacter* sp. RM6137, *Campylobacter* sp. RM12175, *C. showae*, and uncultured *Campylobacter* sp.) displayed higher co-occurrence similarities compared to the blue cluster (*C. rectus, C. subantarcticus, C. hepaticus, C. hominis, C. concisus, C. canadensis, and C. pelondis)* and the violet cluster (*C. avium, C. cuniculorum, C. insulaenigrae, C. volucris*, and *C. troglodytis*), respectively based on dimension 1, which explained 44.4% variability between the cluster. Interestingly, the green cluster was mostly composed of the five most commonly reported thermotolerant *Campylobacter* species (*C. coli, C. jejuni, C. helveticus, C. upsaliensis*, and *C. lari*), while the red cluster consisted of non-thermotolerant *Campylobacter* species (*C. hyointestinalis, C. fetus*, and *C. showae*).

Unlike the PCR data, the distribution of the *Campylobacter* positive children stools (at the genus and species level), and the prevalence of the *Campylobacter* species in the stools were overall independent of the kebeles (*P* > 0.05; Figure 2, Figures S4A,B). Approximately 65% (45–84%) of the stool samples clustered within their own kebeles based on the abundance of the *Campylobacter* spp. detected in the stool samples (Figure S4C). Therefore, due to the high variability observed within most of the kebeles (4/5), the five kebeles were pooled into one (*n* = 100) to enhance the veracity of the MeTRS data described below. Only the abundance (read per million) of *C. hyointestinalis* and *Campylobacter* sp. RM12175 significantly differed between kebeles (*P* < 0.01; Figure S5). Finkle harbored significantly higher level of *C. hyointestinalis* in the stools compared to Gobe Chala, Negeya, and Biftu Geda (Figure S5A); and Damota harbored significantly higher level of *Campylobacter* sp. RM12175 in the stools compared to the other four kebeles (Figure S5B).

Overall, the percentage of *Campylobacter* positive stools was significantly higher using the MeTRS approach compared to the conventional PCR approach (*P* < 0.01; Figures S2A,S2B). Further, some discrepancies were observed between the two methods used (Figure S2C). Out of the 88 stools positive for *Campylobacter* via MeTRS, 42 of them (48%) were also identified positive via conventional PCR (16S genus-specific primers); however, five of the 50 (10%) stools positive for the *Campylobacter* genus via conventional PCR were not positive for the *Campylobacter* via MeTRS analysis (Figure S2C). Similarly, based on the species-specific PCR (*ceuE* and *mapA* primers), six of 13 (46%) and two of two (100%) stools were positive for *C. jejuni* and *C. coli*, respectively only by PCR but not with MeTRS (Figure S2C).

### The *Campylobacter* in the Stools Was Not Associated With Diarrhea and EED Severity

To determine the role of *Campylobacter* in EED, the levels of lactulose and MPO were measured as indicators of EED (24, 25). Additional details concerning the prevalence of EED and stunting among children in this study are described in Chen et al. (under review).

Out of the 100 children studied, 55 children possessed “normal” gut permeability (L% below 0.2), 29 children possessed “moderate” defect in gut permeability (L% between 0.2 and 0.45), and 16 children possessed “severe” defect in gut permeability (L% ratio above 0.45). No correlations were identified when a multivariate analysis was performed between L% and the prevalence or the abundance of *Campylobacter* detected in the children stools (*P* > 0.05). On the other hand, the discriminant analysis showed that the overall *Campylobacter* spp. composition in the stool differed between the L% status (“normal,” “moderate,” and “severe”; *r*^2^ = 0.37; Figure S6A). Eighty two percent (45/55) of the children identified as “normal” clustered together based on the *Campylobacter* composition in the stool. Besides, only 66% (19/29) and 44% (7/16) of the child identified as with “moderate” and “severe” permeability defect, respectively clustered within their own group based on the *Campylobacter* composition in the stool (Figure S6B). Further, 28% (8/29) and 41% (7/17) of the children identified as with “moderate” and “severe” permeability defect clustered with the children identified as “normal.” Overall, the *Campylobacter* composition in the stool samples was associated with 58% of the children with “moderate” and “severe” gut permeability defect based on the L% data.

Similarly, based on the MPO levels, out of the 100 children studied, 43 children possessed “normal” gut ([MPO] below 2,000 ng/ml), 32 children possessed “moderate” gut inflammation ([MPO] between 2,000 and 11,000), and 25 children possessed “severe” gut inflammation ([MPO] above 11,000). No correlations were identified when a multivariate analysis was performed between [MPO] and the prevalence or the abundance of *Campylobacter* detected in the children stools (*P* > 0.05); however, the *Campylobacter* spp. composition in the stool differed between the inflammation status (*r*^2^ = 0.37; Figure S6C). Fifty eight percent (25/43), 88% (28/32) and 48% (12/25) of the children with inflammation status “normal,” “moderate,” and “severe,” respectively clustered within their own group based on the *Campylobacter* composition in the stool (Figure S6D). Only 9% (3/32) and 8% (2/25) of the children identified as having “moderate” and “severe” gut inflammation clustered with the children identified as “normal.” Overall, the *Campylobacter* composition in the stool was associated with 70% of the children with “moderate” and “severe” gut inflammation based on the MPO data.

Because of the lack of correlation between the *Campylobacter* and the L% and MPO, both parameters were combined together to build an index estimating the EED (Figure S1). Out of the 100 children studied, 50 children were classified as “normal,” 33 children having “moderate” EED, and 25 children having “severe” EED (Figure 1). As observed above, the *Campylobacter* spp. composition in the stool differed between the EED status (*r*^2^ = 0.38; Figure S6E). Ninety percent (45/50), 61% (20/33), and 53% (9/17) of the children identified as “normal,” “moderate,” and “severe,” respectively clustered within their own group based on the *Campylobacter* composition in the stool (Figure S6F). Overall, the *Campylobacter* composition in the stool was associated with 58% of the children with “moderate” and “severe” EED based on the EED status data.

In addition, the prevalence of child with diarrhea was recorded to determine the associations with *Campylobacter* spp. prevalence and abundance in the stool samples. Additional details concerning the diarrhea data are described in Chen et al. (under review). Out of the 100 children studied, 48% of the children had diarrhea on the day of the stool collection or 15 days before stool collection (Figure 1). No correlations were identified when a multivariate analysis was performed between the diarrhea data and the prevalence or abundance of *Campylobacter* spp. in the stool samples (*P* > 0.05). However, the discriminant analysis showed that overall the *Campylobacter* spp. composition in the stool differed based on the diarrhea status of the children (*r*^2^ = 0.32; Figure S6G). Eighty-one percent (39/48) of the children with diarrhea clustered together based on the *Campylobacter* composition in the stool samples, while 58% (30/52) of the children without diarrhea clustered together as a separate cluster based on the *Campylobacter* composition in stool (Figure S6H).

### Specific Bacteria of the Stool Microbiome Correlated With the *Campylobacter* Prevalence and EED Severity

Post-filtering, the MeTRS identified a total of 2,353 bacteria, 642 Archaea, 17 virus/viroid, and 249 eukaryotes at the species level among the 100 children stool samples studied. The global analysis of the fecal microbiota revealed that the majority of children (*n* = 89) harbored similar microbiota profile (*P* < 0.01; Figure S7A). Between 68% and 84% of the stool samples clustered by kebeles based on the microbiota composition (Figure S7B). None of the stool samples from Damota and Biftu Geda displayed microbiome composition similarities to Finkle stool samples (Figures S7B,S7C), despite the fact that Finkle is geographically closer to Damota and Biftu Geda compared to the other kebeles (Figure 1). Further, 19 stools samples displayed higher microbiome composition similarities with Biftu Geda or Negeya stool samples compared to the original kebeles (Figures S7B,S7C), independently of the distance between the kebeles (Figure 1). Given the discriminant analysis showed that the ellipses (CI 95%) of most kebeles (4/5) were overlapping, the MeTRS data were analyzed as one population (*n* = 100). No distinct correlation was detected between the *Campylobacter* species and the virus/viroid and eukaryotes (*P* > 0.05, *r*^2^ < 0.25); by consequence, the majority of the results described below focused on the interconnections between *Campylobacter* spp. and stool bacterial community.

Our analysis showed that the bacterial composition in the stool samples was related to the overall presence of *Campylobacter* spp. (Figure S8A). Ninety-four percent (83/94) of the children stools positive for *Campylobacter* spp. clustered together based on their microbiome composition (Figure S8B). Only 58% (7/12) of the child stools negative for *Campylobacter* spp. clustered together in a separate cluster based on their microbiome composition. Approximately 16.9% of the bacteria identified in stool samples (*n* = 387/2,353) were positively or negatively correlated with the prevalence or abundance of *Campylobacter* in the stool samples (*r*^2^ > 0.2 or *r*^2^ < −0.20; *P* < 0.05). Among the bacteria correlated with *Campylobacter*, several of them belonged to *Arcobacter, Bacillus, Bacteroides, Bifidobacterium, Capnocytophaga, Clostridium, Collinsella, Corynebacterium, Enterobacter, Enterococcus, Helicobacter, Lactobacillus, Olsenella, Paenibacillus, Pantoea, Prevotella, Serratia, Streptococcus*, and *Veilonella*.

**Figure 4:**
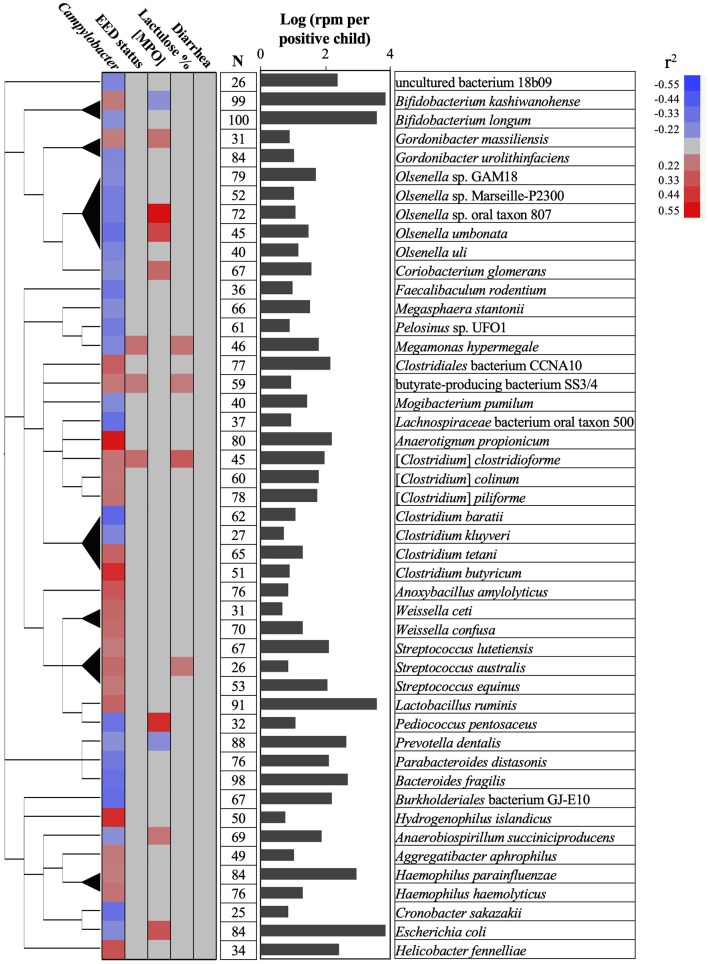
Associations between *Campylobacter*, gut permeability, gut inflammation, environmental enteric dysfunction (EED), diarrhea, and child stool microbiome. A total of 47 bacterial species positively or negatively correlated (red or blue cells, respectively) with *Campylobacter* prevalence and abundance (reads per million, rpm; *r*^2^ > 0.20 or *r*^2^ < −0.20; *P* < 0.05), gut permeability (lactulose %), [MPO] in ng/ml, EED status (“normal,” “moderate” EED, “severe” EED), and/or the diarrhea prevalence data. Additional details concerning the EED severity determination are presented in Table S1. The phylogenetic tree was built using NCBI website (https://www.ncbi.nlm.nih.gov/Taxonomy/CommonTree/wwwcmt.cgi). N: number of stools positive for the designated bacterial species. The bar graph represents the average abundance (log rpm per positive stool sample) for the selected bacterial species. rpm, read per million; MPO, myeloperoxidase.

However, out of the 387 species mentioned above only 47 were correlated (25 negatively and 22 positively) with the prevalence or abundance of *Campylobacter* in the stool samples (*r*^2^ > 0.2 or *r*^2^ < −0.2; *P* < 0.05) and detected in at least 25 children stool samples (average of 60 children; Figure 4). *Olsenella, Clostridium*, and *Streptococcus* were the most represented genus in this subset. *Anaerotignum propionicum* (*r*^2^ = 0.53), *Clostridium butyricum* (*r*^2^ = 0.47)*, Hydrogenophilus islandicus* (*r*^2^ = 0.46) displayed the highest positive correlation with *Campylobacter* abundance in the stool samples; while *Burkholderiales* bacterium GJ-E10 (*r*^2^ = −0.36)*, Pasteurellaceae* bacterium NI1060 (*r*^2^ = −0.35), and *Clostridium baratii* (*r*^2^ = −0.38), displayed the highest negative correlation with *Campylobacter* abundance and/or prevalence in the stool samples.

Only *Prevotella dentallis* (2.63-log rpm per positive stool sample) was negatively correlated with both the *Campylobacter* abundance and/or prevalence in the stool samples (*r*^2^ = −0.2) and the [MPO] (*r*^2^ = −0.22). On the other hand, Butyrate-producing bacterium SS3/4, [*Clostridium*] *clostridioforme*, and *Streptococcus australis* were positively correlated with both the *Campylobacter* abundance and/or prevalence in the stool (*r*^2^ > 0.2) and at least with one of the following parameters ([MPO], lactulose%, and/or EED severity; *r*^2^ > 0.21). *Anaerobiospirillum succiniciproducens, Coriobacterium glomerans, Escherichia coli, Megamonas hypermegale, Olsenella* sp. oral taxon 807, *Olsenella umbonata*, and *Pediococcus pentosaceus* were negatively correlated with the *Campylobacter* abundance and/or prevalence in the stool (*r*^2^ < -0.2) but positively correlated with at least one of the following parameters ([MPO], lactulose%, and/or EED severity; *r*^2^ > 0.23). Only *Bifidobacterium kashiwanohense* was positively correlated with the *Campylobacter* abundance and/or prevalence in the stool (*r*^2^ > 0.21) and negatively correlated with [MPO] (*r*^2^ < −0.2).

No bacteria correlated with *Campylobacter* spp. were correlated with the presence or absence of diarrhea.

### Specific Bacteria of the Stool Microbiome Correlated With the Gut Permeability, Gut Inflammation, EED Severity, and Diarrhea in Children

The microbiome composition was related to the gut permeability (L%), gut inflammation (MPO), and EED status (Figure S9B), as observed with the *Campylobacter* composition in the stool samples (Figure S6); however, higher correlations between the L%, EED status and the microbiome composition were observed compared to the correlation obtained with *Campylobacter*. Out of the 100 children studied, 80% (44/55), 79% (23/29), and 81% (13/16) of the children identified as “normal,” “moderate,” and “severe” based on the L%, respectively, clustered within their own group based on the microbiome composition (Figures S9A,S9B). Eighty one percent (35/43), 72% (23/32), and 68% (17/25) of the children identified as “normal,” “moderate,” and “severe” based on MPO levels, respectively, clustered within their own group based on the microbiome composition in the stool (Figures S9C,S9D). Eighty percent (40/50), 79% (26/33), and 76% (13/17) of the children identified as “normal,” “moderate,” and “severe” based on the EED status (both L% and MPO combined; Table S1), respectively, clustered within their own group based on the microbiome composition in the stool (Figures S9E,S9F). Overall, the microbiome composition was associated with 80% of the gut permeability data, 70% of the gut inflammation data, and 78% of the EED severity data observed in the children with “moderate” and “severe” status. Similarly, children with diarrhea displayed different microbiome composition compared to children with no diarrhea (Figure S9G). Out of the 100 children, 81% (42/52) of the stools from children with no diarrhea and 85% (41/48) of the stools from children with diarrhea clustered to their respective groups based on the microbiome composition (Figure S9H).

A total of 49 bacteria not-associated with *Campylobacter* were correlated (30 positively [*r*^2^ > 0.21; *P* < 0.05] and 19 negatively [*r*^2^ < −0.20; *P* < 0.05]) with the L%, MPO levels, and/or the EED severity and detected in at least 25 children stool samples (Figure S9I). Only *Neisseria elongata* was positively correlated with the EED severity (*r*^2^ = 0.31; 2.2-fold increased between the “normal” children and the children with “severe” EED) and also L% (lactulose%; *r*^2^ = 0.21; 1.7-fold increased between the children with “normal” and “severe” gut permeability). Eight bacteria (*Lachnoclostridium* sp. YL32, [*Clostridium*] *bolteae, Bifidobacterium bifidum, Mordavella* sp. Marseille-P3756, *Bacteroides thetaiotaomicron, Streptococcus salivarius*, and *Libanicoccus massiliensis*) highly prevalent (>71%) and abundant (>2.0-log rpm per stool sample in children with “severe” status) in the stools were positively correlated (*r*^2^ between 0.24 and 0.36) with L% or MPO levels. On the other hand, *Lactobacillus mucosae* and *Pasteurella multocida* were less prevalent (39 and 60%, respectively) and less abundant (below 1.0 and 1.5-log rpm per stool, respectively) in the stools, but they were higher (2.5- and 2-fold, respectively) in the children with “severe” gut inflammation compared to the “normal” children.

Five bacteria (*Mogibacterium diversum, Ethanoligenens harbinense, Roseburia hominis, Ruminococcus* sp. SR1/5, and *Bacteroides dorei*) were negatively correlated with the EED severity (*r*^2^ between −0.21 and −0.27) and detected in at least 40% of the children stools (Figure S9I). Interestingly, only *M. diversum* was negatively correlated with all the parameters studied (EED severity [*r*^2^ = −0.27], L% [*r*^2^ = −0.20], MPO [*r*^2^ = −0.26], and diarrhea prevalence [*r*^2^ = −0.25]). Further, *M. diversum* abundance in stools was higher in the “normal” children compared to the children with “severe” EED (2.7-fold), with “severe” L% (4-fold), and with diarrhea (2-fold). Similarly, *E. harbinense* was higher (2.3-fold) in the stools of “normal” children compared to the children with “severe” EED. Four bacteria (*Clostridium taeniosporum, Hungatella hathewayi, Selenomonas ruminantium*, and *Cryotobacterium curtum*) were less prevalent (between 40% and 58%) and abundant (<1.3-log rpm per stool) in the stools, but they were detected at higher level (2.2-, 2-, 2.6-, and 2-fold, respectively) in the stools of “normal” children compared to the children with “severe” MPO. Further, *Selenomonas ruminantium* was also detected at higher level (2-fold) in children with no diarrhea compared to the ones with diarrhea.

Three additional bacteria (*Prevotella scopos, S. ruminantium*, and *Measphaera els*) highly prevalent (>79%) and abundant (>2.1-log rpm per stool) in the stools and not-associated with *Campylobacter* were negatively correlated (*r*^2^ between −0.2 and −0.22) with the diarrhea data (Figure S9I).

## Discussion

The purpose of this study was to estimate the prevalence, diversity, abundance and co-occurrence of *Campylobacter* in the stool of young children from Eastern Ethiopia and assess potential associations between *Campylobacter* and diarrhea and EED (31). To accomplish this task, the use of traditional microbiology approaches to isolate thermophilic *Campylobacter* spp. was attempted; however, due to technical and logistical challenges faced in Ethiopia, the results generated were deemed unreliable (data not shown). The fastidious nature of *Campylobacter* and difficulty of isolation, rendered culture and isolation unreliable for estimation of prevalence (32). By consequence, other studies utilized different approaches to estimate the prevalence of *Campylobacter* in stool samples, including enzyme immunoassay (EIA), PCR, and shotgun metagenomics (7, 33, 34). Two different culture independent approaches (conventional PCR and MeTRS) were used in this study to estimate the prevalence of *Campylobacter* in the child stool samples.

The number of child stools detected positive for the *Campylobacter* genus was significantly higher using the MeTRS approach (88% [80–93.6%]) compared to the genus-specific PCR approach (50% [40–61%]; 16S RNA primer). Several studies showed that shotgun metagenomics and MeTRS had at least equal sensitivity to real-time PCR based approach for different pathogens (29, 35–37). Further, discrepancies between the two detection methods was observed, especially with the species-specific PCR approach (*ceuE* primer for *C. coli* and *mapA* primer for *C. jejuni*). However, the reason for this discrepancy is unknown and needs further investigation. Many factors could affect the sensitivity of both PCR and MeTRS approaches, including the type of the sample, the quality and quantity of the extracted DNA, the agreement between the method used, the type of the organism under investigation, and the tools used to analyze the data. A recent study suggested that shotgun metagenomics might overestimate the prevalence of microbial population due to the presence of plasmid sequences within the assembled genome and the high sequences similarities between bacterial species (38). In our study, by taking an approach of sequencing the total RNA component, we are able to better identify species rather than by sequencing DNA alone. However, the genome coverage obtained with the MeTRS approach was not high enough to provide information about strain relatedness within the same species. Therefore, metagenomic studies using long read technologies or whole genome sequencing of pure isolates are still needed for source attribution purposes.

It is commonly known that *C. jejuni* and *C. coli* are leading causes of campylobacteriosis in the western world (39); however, our study revealed that most of the children stools positive for *Campylobacter* (94%) harbored more than one *Campylobacter* species. This finding suggests that *Campylobacter* colonization of children may have occurred through multiple reservoirs or from a reservoir in which several *Campylobacter* species may co-inhabit. This hypothesis is supported by the existing literature which describes colonization of multiple hosts by a given *Campylobacter* species; for example, *C. hyointestinalis* is commonly found in swine, sheep, dog, and in cattle (40). Further, twelve *Campylobacter* spp. (*Campylobacter* sp. RM12175, *C. hyointestinalis, Campylobacter* sp. RM6137, uncultured *Campylobacter* sp*., C. upsaliensis, Campylobacter* sp. NCTC 13003*, C. helveticus, C. lanienae, C. concisus, C. fetus, C. pinnipediorum*, and *C. showae*) in addition to *C. jejuni* and *C. coli* were detected in at least 40% of the stool samples at relatively high abundance. Our study also showed that several *Campylobacter* species were commonly co-occurring within the same stool samples, while others had opposite trends. However, none of the clusters representing different *Campylobacter* species (Figure 3) were correlated with the gut permeability, gut inflammation, EED severity, and diarrhea (data not shown). These facts raise many questions about the mechanisms underlying the *Campylobacter* species diversity observed and their potential effect on gut health and children's growth. A possible contributor to this diversity could be the diverse environment surrounding these children. A previous study demonstrated significant associations between *Campylobacter* isolated from children (under 5 years of age) and the exposure to domestic animal (pets, chickens, and pigeons) in Gondar, North Western Ethiopia (15). It was estimated that approximately 83% of the rural households in Ethiopia possess livestock (cattle, goat, and sheep and chicken) (23). Further, most of these *Campylobacter* species mentioned above (except *Campylobacter* sp. RM6137) were reported in livestock such as cattle, sheep, and goat (41). Therefore, livestock most likely plays a major role as reservoir and in the horizontal transmission of *Campylobacter* to children in Ethiopia. Hence, concurrently studying the prevalence and diversity of *Campylobacter* in livestock and children would provide key information concerning the horizontal transmission of *Campylobacter* between livestock and children. It is also important to notice that four of the most prevalent *Campylobacter* species (*C. hyointestinalis, C. helveticus, C. fetus*, and *C. upsaliensis*) detected in the children stools in this study have not been commonly reported in previous studies, compared to other species such as *C. jejuni, C. coli, C. fetus, C. showae*, and *C. concisus*, which have been associated with human disease (41, 42). The reservoirs of non-thermotolerant *Campylobacters* are very diverse and not well-understood especially in low to middle income countries.

Among all the *Campylobacter* species detected in our study (27 classified species and 12 unclassified species), the non-thermotolerant *C. hyointestinalis* was the second most abundant and the third most prevalent *Campylobacter* species in the children stools. *C. hyointestinalis* was previously isolated from numerous livestock species such as swine (43), cattle (43–48), sheep (43). However, its implication in human diseases is rare and sporadic (49–53). *C*. *hyointestinalis* has also been reported in developed countries, more precisely in feces collected from dairy farms in Canada (19.3%), in Finland (10.8%) and from dairy goat farms in New Zealand (2%), and in 3.2% milk samples collected from dairy farms in Italy (45, 47, 48, 54). *C. hyointestinalis*, as well as, other *Campylobacter* species were also isolated from wild boar in Japan and dogs in Canada (55, 56). By consequence, studying the prevalence, diversity, and abundance of *Campylobacter* species in livestock, also in domestic- and wild animals that have a high likelihood of interacting with livestock or humans is essential to understand the transmission dynamics of *Campylobacter* to children.

Overall, the MeTRS data showed that the microbial population in the stools in different kebeles was homogeneous between the children. No correlations between *Campylobacter* spp. and gut permeability, gut inflammation, EED severity, or diarrhea status were observed in our study; however, characteristic stool microbiome composition profiles were detected based on the prevalence and abundance of *Campylobacter* spp. in the stools, gut permeability, gut inflammation, EED severity, and diarrhea status. Up to 0.9% (*n* = 22 bacterial species) of the microbiome was positively correlated (*r*^2^ > 0.20) and 1.1% (*n* = 25 bacterial species) was negatively correlated (*r*^2^ < −0.21) with the prevalence and/or abundance of *Campylobacter* spp. in the stools. Among them, only *Prevotella dentalis* was highly prevalent (88%) and abundant (2.63-log rpm per stool) in the stools, and negatively correlated with both *Campylobacter* prevalence (*r*^2^ = −0.2) and the gut inflammation (*r*^2^ = −0.21). *Prevotella* is associated with plant-rich diets and also promotes chronic inflammation (57, 58). *Prevotella* is known to provide key nutrients to other bacteria of the human microbiota and may act on the pathogenicity of specific pathobionts (59–63); however, no studies demonstrated yet potential antagonistic interactions between *P. dentalis* and *Campylobacter*.

*Bifidobacterium kashiwanohense* was highly prevalent (99%) and abundant (3.84-log rpm per stool) in the stools and was also negatively correlated with gut inflammation (*r*^2^ = −0.2). This bacterium possesses high iron sequestration properties and might produce indole-3-lactic acids, which was shown to be a successful strategy to inhibit and compete with enteric pathogens (i.e., *Salmonella* and *E. coli*) (64, 65). However, *B. kashiwanohense* was also positively correlated (*r*^2^ = 0.21) with *Campylobacter* in our study, suggesting that the modulation of *B. kashiwanohense* may reduce the impact of pathobiont on the intestinal homeostasis but facilitates the persistence of *Campylobacter* in the child intestinal tract. On the other hand, *Bifidobacterium longum* was negatively correlated with *Campylobacter* in our study, which concords with previous published studies (66). Similarly, other bacteria (*Gordonibacter urolithinfaciens, Mogibacterium pumilum, Megasphaera stantonii, E. coli, Clostridium* spp*., Olsenella* spp., and *Bacteroides fragilis*) were negatively correlated with *Campylobacter* in the stools. Several studies showed that *Bacteroides, Gordonibacter*, and *Escherichia* produce molecules with antimicrobial and anti-inflammatory properties (lactic acids, short chain fatty acids, enterocins, and urolithins), which could modulate the microbiome quality, intestinal homeostasis, and host immune responses and could be effective against *Campylobacter* (67–74). Interestingly, several *Olsenella* spp. (*n* = 5) were frequently detected in the stool samples and were negatively correlated with *Campylobacter* prevalence. *Olsenella* was previously identified as a part of the microbiome associated with the infant health status (stunting, autism spectrum disorder, and chronic malnutrition) in China and Bangladesh (75–77). Therefore, the anti-*Campylobacter* properties of several species identified in this study and their potential application as dietary supplement to control *Campylobacter* in developing countries needs further investigation.

Interestingly, *M. diversum* was the only bacteria of the stool microbiome with a distinct positive effect on the child gut permeability (*r*^2^ = −0.2), gut inflammation (*r*^2^ = −0.26), EED severity (*r*^2^ = −0.27), and diarrhea prevalence (*r*^2^ = −0.24), but no association with *Campylobacter*. No information is available concerning this bacterium. Therefore, further investigations are required to support the beneficial properties of *M. diversum* against EED and their potential application as dietary supplement in developing countries to improve intestinal homeostasis. On the other hand, the abundance of four bacterial species (butyrate-producing bacterium SS3/4, *Megamonas hypermegale, Streptococcus australis*, and *Gordonibacter massiliensis*) were positively correlated with the abundance of *Campylobacter* in the stools and with the severity of the gut inflammation, gut permeability and/or EED severity; Therefore, these bacteria might have an important impact on the gut microbiome quality, the persistence of *Campylobacter* in the gut, and the children's health.

In conclusion, we report in this study high prevalence and diversity of *Campylobacter* spp. in children in eastern Ethiopia, including the non-thermophilic *Campylobacter* spp. (i.e., *C. hyointestinalis* and *C. fetus-*like species). The MeTRS revealed co-occurrence of several *Campylobacter* spp. More studies are needed to better understand the prevalence, sources, and transmission dynamics of *Campylobacter* to children as well as to establish a clear link between *Campylobacter* infection (including non-thermotolerant species) in children with EED and stunting. Furthermore, even though no direct correlation was identified between *Campylobacter* prevalence and abundance, and the EED severity or the diarrhea, the metagenomic analysis highlighted the association between stool microbiome and the prevalence and abundance of *Campylobacter* spp., EED severity, and diarrhea in children. The unique changes in microbiome may serve as biomarkers for disease status and can help to elucidate the complex interactions between *Campylobacter*, the gut microbiome, EED and stunting in the context of the socio-economic environment of children in rural Ethiopia.

## Data Availability Statement

The datasets generated for this study can be found in the BioProject ID: PRJNA608948 https://www.ncbi.nlm.nih.gov/sra/?term=SRR11194563.

## Ethics Statement

All the study procedures were performed in accordance with the Declaration of Helsinki (78) and approvals from the HU Institutional Health Ethics Research Review Committee (Ref. No. IHRERC/152/2018), the Ethiopia National Research Ethics Review Committee (Ref. No. MoST/3-10/168/2018), the Institutional Review Board at the University of Florida (UF) (Ref. No. 201703252) and Washington University School of Medicine (Protocol No. 201806021). Additionally, the operational district officers and local village chiefs were informed about the objectives of the study and the future impacts and their consent was obtained. Written and oral consents were also obtained from child's mother/caregiver and father. Material and Data Transfer Agreements (MDTA) were signed between Haramaya University and all US-based partners. Export permits to ship biological specimens from Ethiopia to the U.S.A were approved by the Ministry of Science and Higher Education (Ref. No. SHE/SSM/19.1/008/11/19). Written informed consent to participate in this study was provided by the participants' legal guardian/next of kin.

## Author Contributions

GR, SM, JY, WG, MM, AH, and LD conceived and designed the experiment. YT, AM, and GY collected samples. YT, LD, MG, YH, BM, VA, MO, and KK processed the samples for PCR and MeTRS analyses. YT, LD, AH, YH, GR, NS, MG, VA, KK, and DC analyzed the PCR and MeTRS data. YT, LD, MG, AH, and GR wrote the manuscript.

### Conflict of Interest

The authors declare that the research was conducted in the absence of any commercial or financial relationships that could be construed as a potential conflict of interest.

## Supplementary Material

Pipeline used for the processing of the MeTRS data.

Figure S1 Estimation of environmental enteric dysfunction severity based on gut permeability and gut inflammation data. The gut permeability was assessed using the percentage of lactulose in the urine. The gut inflammation was assessed based on the concentration in MPO (ng/ml) in the urine. Both parameters were used to estimate the environmental enteric dysfunction (EED) severity for each infant. The gut inflammation was considered normal if [MPO] was lower than 2,000 ng/ml, moderated if [MPO] was between 2,000 and 11,000 ng/ml, and severe if [MPO] was higher than 11,000 ng/ml. The gut permeability was considered normal if the lactulose value was lower than 0.2%, moderated if the lactulose value was between 0.2 and 0.45%, and severe if the lactulose value was higher than 0.45%; MPO, myeloperoxidase.

Figure S2 *Campylobacter* spp. prevalence in the children stools (*n* = 100) collected from five kebeles using conventional PCR **(A)** and MeTRS **(B)** data. Orange and blue bars represent the percentage of the stools positive or negative, respectively for *Campylobacter* for a given kebele. C) Comparison of the *Campylobacter* prevalence using conventional PCR (c-PCR) and meta-total RNA sequencing (MeTRS). In red and blue are the stools positive or negative, respectively for *Campylobacter*. The heat map is composed of 100 columns (one column per child). Genus-specific PCR was performed using 16S RNA primers and species-specific PCR for *C. coli* and *C. jeju*ni were performed using *ceuE* and *mapA* primers, respectively (Table S1).

Figure S3 Co-occurrence of *Campylobacter* spp. in the children stool samples. Co-occurrence Heatmap profile was created using the multivariate analysis data (*r*^2^) based on the prevalence of *Campylobacter* spp. in the stool samples (see Figure 2).

Figure S4 *Campylobacter* spp. profile between children stool samples based on the meta-total RNA sequencing (MeTRS). **(A)** Principal component analysis of the *Campylobacter* spp. diversity and abundance in the stools. Each colored dot represents one child stool sample (*n* = 100). Black dot represents the average profile for a given kebele (*n* = 5). **(B)** Discriminant analysis of the *Campylobacter* spp. diversity and abundance in the stools based on kebeles. The outside ellipse contains ~50% of the observations. The inside ellipse represents 95% confidence level (Cut-off for MeTRS data; contigs number ≥ 10; Read length ≥ 50; Z-score ≥ 1. **(C)**. Clusterization profile of the stools based on the *Campylobacter* spp. diversity, prevalence, and abundance data.

Figure S5 Differences in abundance of *Campylobacter hyointestinalis and Campylobacter* RM12175 between kebeles. Letters **(A,B)** indicates different statistical groups (*P* < 0.01). Star: read per million (rpm) are significantly higher in the designated kebele compared to the other kebeles.

Figure S6 *Campylobacter* spp. profiles between children stool samples based EED severity and diarrhea. Discriminant analysis of the *Campylobacter* spp. abundance in the children stools based on **(A)** the gut permeability (lactulose%), **(C)** gut inflammation ([myeloperoxidase] in ng/ml), **(E)** EED severity, and **(G)** diarrhea prevalence data. The outside ellipse contains approximately 50% of the observations. The inside ellipse represents 95% confidence level. Clusterization profile of the *Campylobacter* spp. abundance in the stools based on **(B)** the gut permeability (lactulose%), **(D)** gut inflammation ([myeloperoxidase] in ng/ml), **(F)** EED severity, and **(H)** diarrhea prevalence data. Additional details concerning the EED severity determination are presented in Table S1.

Figure S7 Microbiome profiles between children stool samples based on kebeles. **(A)** Principal component analysis of the children stool samples based on kebeles. Each colored dot represents one stool sample (*n* = 100). Kebeles with the same color code belong to the same cluster and therefore harbored equivalent microbiota diversity and abundance. A *T*^2^ test revealed a total of 11 outliers (stools with significantly different microbiota profile compared to the rest of the population; outside the red oval). **(B)** Discriminant analysis of the stool microbiome based on kebeles. The outside ellipse contains ~50% of the observations. The inside ellipse represents 95% confidence level. **(C)** Clusterization profile of the stool microbiome based on kebeles.

Figure S8 Microbiome profile based on *Campylobacter* prevalence. **(A)** Discriminant analysis of the children stool microbiome based on *Campylobacter* prevalence. The outside ellipse contains ~50% of the observations. The inside ellipse represents 95% confidence level. **(B)** Clusterization profile of the stool's microbiome based on *Campylobacter* prevalence.

Figure S9 Microbiome profile based on environmental enteric dysfunction (EED). Discriminant analysis of the children stool microbiome composition based on **(A)** the gut permeability (lactulose%), **(C)** gut inflammation ([MPO] in ng/ml), **(E)** EED severity, and **(G)** diarrhea prevalence data. The outside ellipse contains ~50% of the observations. The inside ellipse represents 95% confidence level. Clusterization profile of the *Campylobacter* spp. abundance in the stools based on **(B)** the gut permeability (lactulose%), **(D)** gut inflammation ([MPO] in ng/ml), **(F)** EED severity, and **(H)** diarrhea prevalence data. Additional details concerning the EED severity determination are presented in Table S1. **(I)** Correlation between the specific bacterial species of the children stool microbiome and the gut permeability (lactulose%), gut inflammation ([MPO] in ng/ml), EED severity, and diarrhea prevalence data. N, number of stools positive for the designated bacterium. Values in the table represent the mean (log [read per million]) ± standard error for the designated bacterial species for a given status. Stars represent bacterial species harboring at least 2-fold difference in abundance between the “normal” and “severe” status for at least one of the parameters studied. MPO: myeloperoxidase.

Table S1 Primers used in the study. Source: Adapted from Deals et al. (28).
