# Supplementary material for: Co-occurrence of Campylobacter Species in Children From Eastern Ethiopia, and Their Association With Environmental Enteric Dysfunction, Diarrhea, and Host Microbiome
Source: Front Public Health. 2020 Apr 15;8:99. doi: 10.3389/fpubh.2020.00099 (PMC7174729; doi:10.3389/fpubh.2020.00099)
Supplement: Supplementary file 1 — Pipeline used for the processing of the MeTRS data. [file Data_Sheet_1.PDF]

Supplemental Material 1: Pipeline used for the processing of the MeTRS data

```
# STAR Host Filtering
STAR
--outFilterMultimapNmax 99999
--outFilterScoreMinOverLread 0.5
--outFilterMatchNminOverLread 0.5
--outReadsUnmapped Fastx
--outFilterMismatchNmax 999
--outSAMmode None
--clip3pNbases 0
--runThreadN 32
--genomeDir
--readFilesIn [input_files]
--quantMode GeneCounts


# Trimmomatic adapter removal
java -jar /usr/local/bin/trimmomatic-0.38.jar
PE
-phred33
[input_files]
[output_files]
ILLUMINACLIP:illumina_TruSeq3-PE-2_NexteraPE-PE.fasta:2:30:10:8:true
MINLEN:35


# PriceSeqFilter quality filtering
PriceSeqFilter
-a 12
-rnf 90
-log c
-fp [input_files]
-op [output_files]
-rqf 85 0.98


# CD-HIT-DUP duplicate compression
cd-hit-dup
-i [input_files]
-o [output_files]
-e 0.05
-u 70
-i2 [input_files]
```

```
-o2 [output_files]


# Bowtie2 Host Removal
bowtie2
-q
-x {genome_basename}
-f
--very-sensitive-local
-S {output_sam_file}
--seed random_seed
-p 32
-1 [input R1]
-2 [input R2]


# GSNAP Human Removal
gsnapl
-A sam
--batch=0
--use-shared-memory=0
--gmap-mode=all
--npaths=1
--ordered
-t 32
--max-mismatches=40
-D {gsnap_base_dir}
-d {gsnap_index_name}
-o {output_sam_file}
{input_fas}


# GSNAP Alignment to NCBI NT
gsnapl
-A m8
--batch=0
--use-shared-memory=0
--gmap-mode=none
--npaths=100
--ordered
-t 48
--max-mismatches=40
-D {remote_index_dir}
-d nt_k16
{remote_input_files} > {multihit_remote_outfile}
```

```
# Rapsearch2 Alignment to NCBI NR
rapsearch
-d {remote_index_dir}/nr_rapsearch
-e -6
-l 10
-a T
-b 0
-v 50
-z 24
-q {remote_input_files}
-o {multihit_remote_outfile}

# Assembly
spades.py
-1 {input_fasta}
-2 {input_fasta2}
-o {assembled_dir}
-m {memory}
-t 32
—only-assembler


# mapping original reads back to assembled contigs
bowtie2
-x {bowtie_index_path}
-f
-U {fasta_file}
--very-sensitive
-p 32 > {output_bowtie_sam}


# BLAST of assembled contigs to sub-set database
blast_command
-query {assembled_contig}
-db {blast_index_path}
-out {blast_m8}
-outfmt 6
-num_alignments 5
-num_threads 32
```
