# Supplementary figures and images for: Co-occurrence of Campylobacter Species in Children From Eastern Ethiopia, and Their Association With Environmental Enteric Dysfunction, Diarrhea, and Host Microbiome
Source: Front Public Health. 2020 Apr 15;8:99. doi: 10.3389/fpubh.2020.00099 (PMC7174729; doi:10.3389/fpubh.2020.00099)

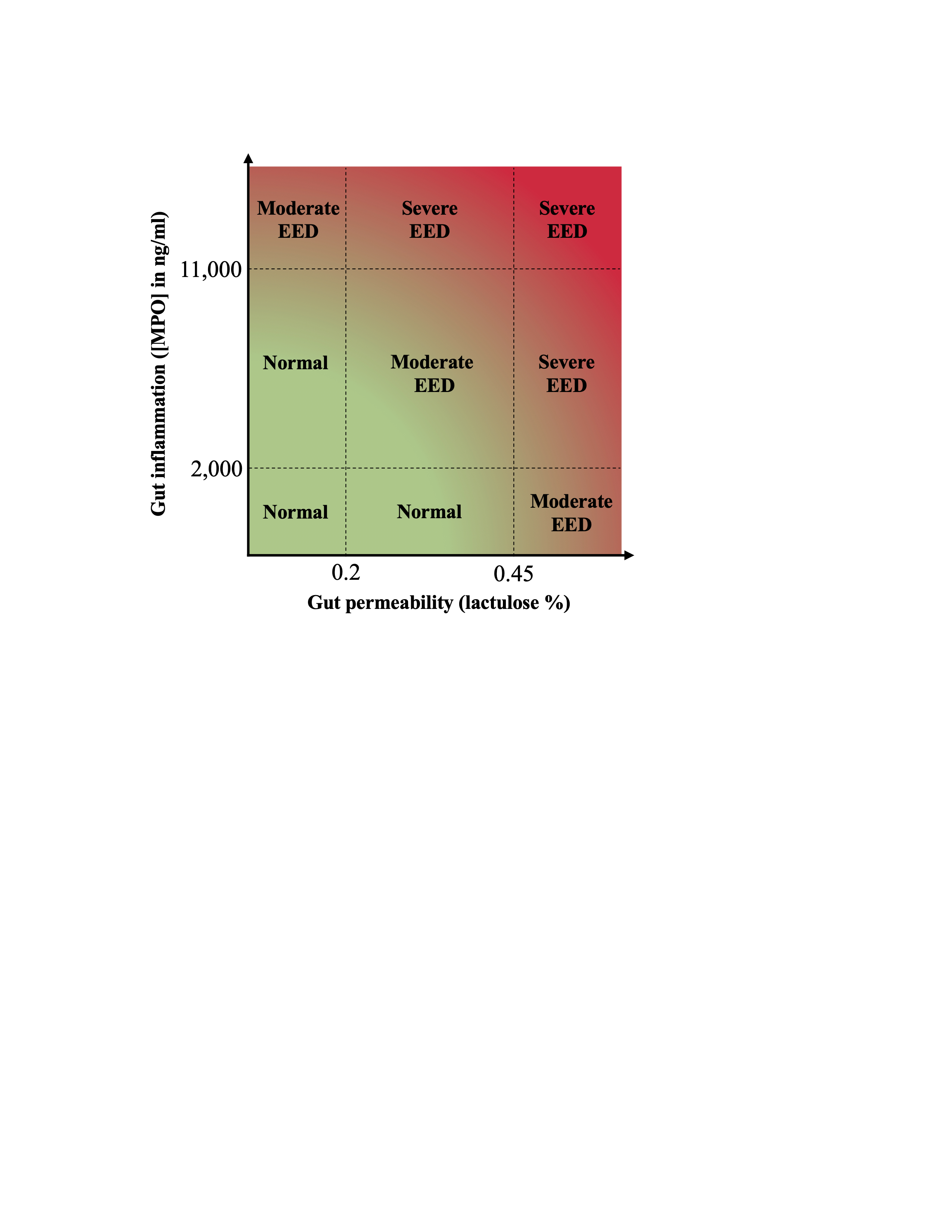

Supplement: Figure S1 — Estimation of environmental enteric dysfunction severity based on gut permeability and gut inflammation data. The gut permeability was assessed using the percentage of lactulose in the urine. The gut inflammation was assessed based on the concentration in MPO (ng/ml) in the urine. Both parameters were used to estimate the environmental enteric dysfunction (EED) severity for each infant. The gut inflammation was considered normal if [MPO] was lower than 2,000 ng/ml, moderated if [MPO] was between 2,000 and 11,000 ng/ml, and severe if [MPO] was higher than 11,000 ng/ml. The gut permeability was considered normal if the lactulose value was lower than 0.2%, moderated if the lactulose value was between 0.2 and 0.45%, and severe if the lactulose value was higher than 0.45%; MPO, myeloperoxidase. [file Image_1.JPEG]

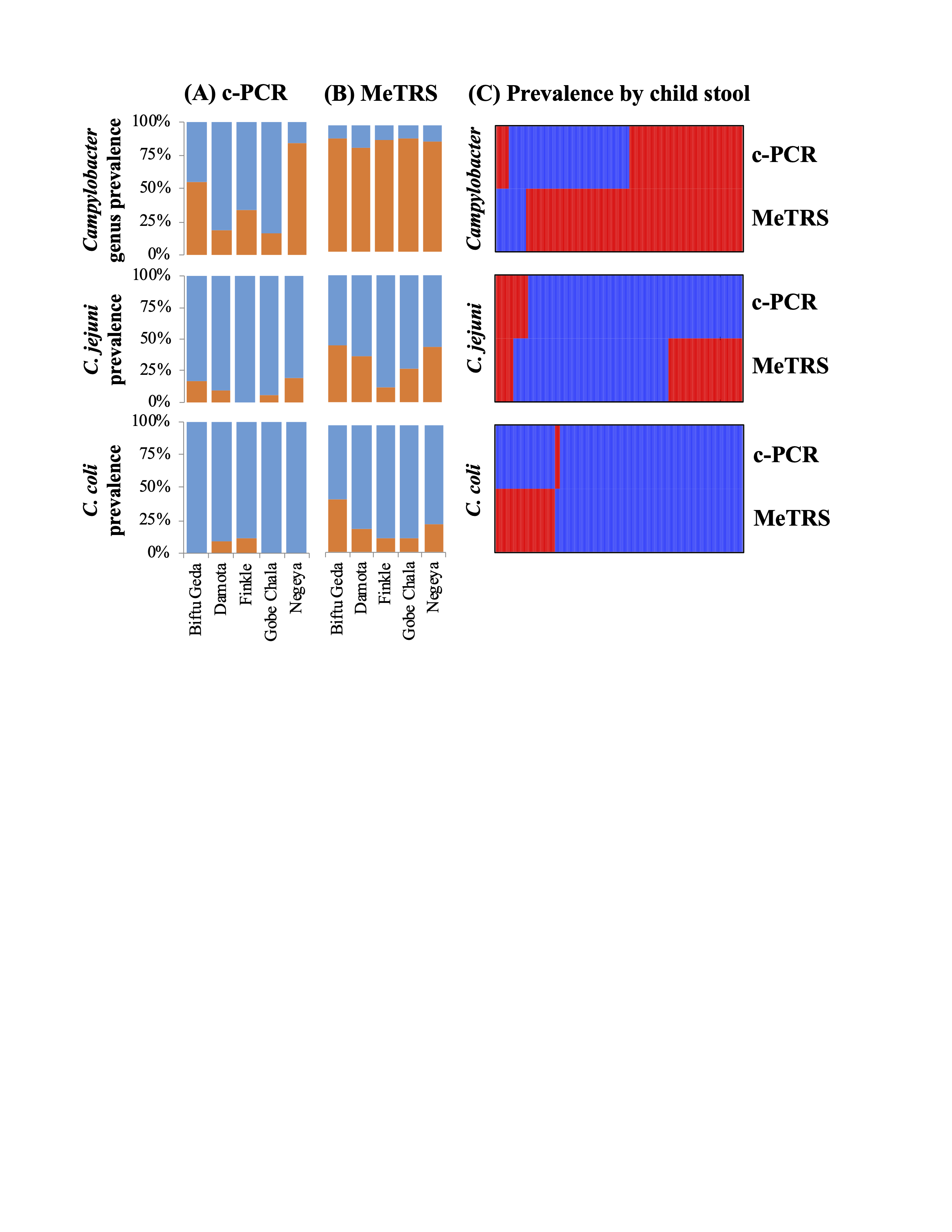

Supplement: Figure S2 — Campylobacter spp. prevalence in the children stools (n = 100) collected from five kebeles using conventional PCR (A) and MeTRS (B) data. Orange and blue bars represent the percentage of the stools positive or negative, respectively for Campylobacter for a given kebele. C) Comparison of the Campylobacter prevalence using conventional PCR (c-PCR) and meta-total RNA sequencing (MeTRS). In red and blue are the stools positive or negative, respectively for Campylobacter. The heat map is composed of 100 columns (one column per child). Genus-specific PCR was performed using 16S RNA primers and species-specific PCR for C. coli and C. jejuni were performed using ceuE and mapA primers, respectively (Table S1). [file Image_2.JPEG]

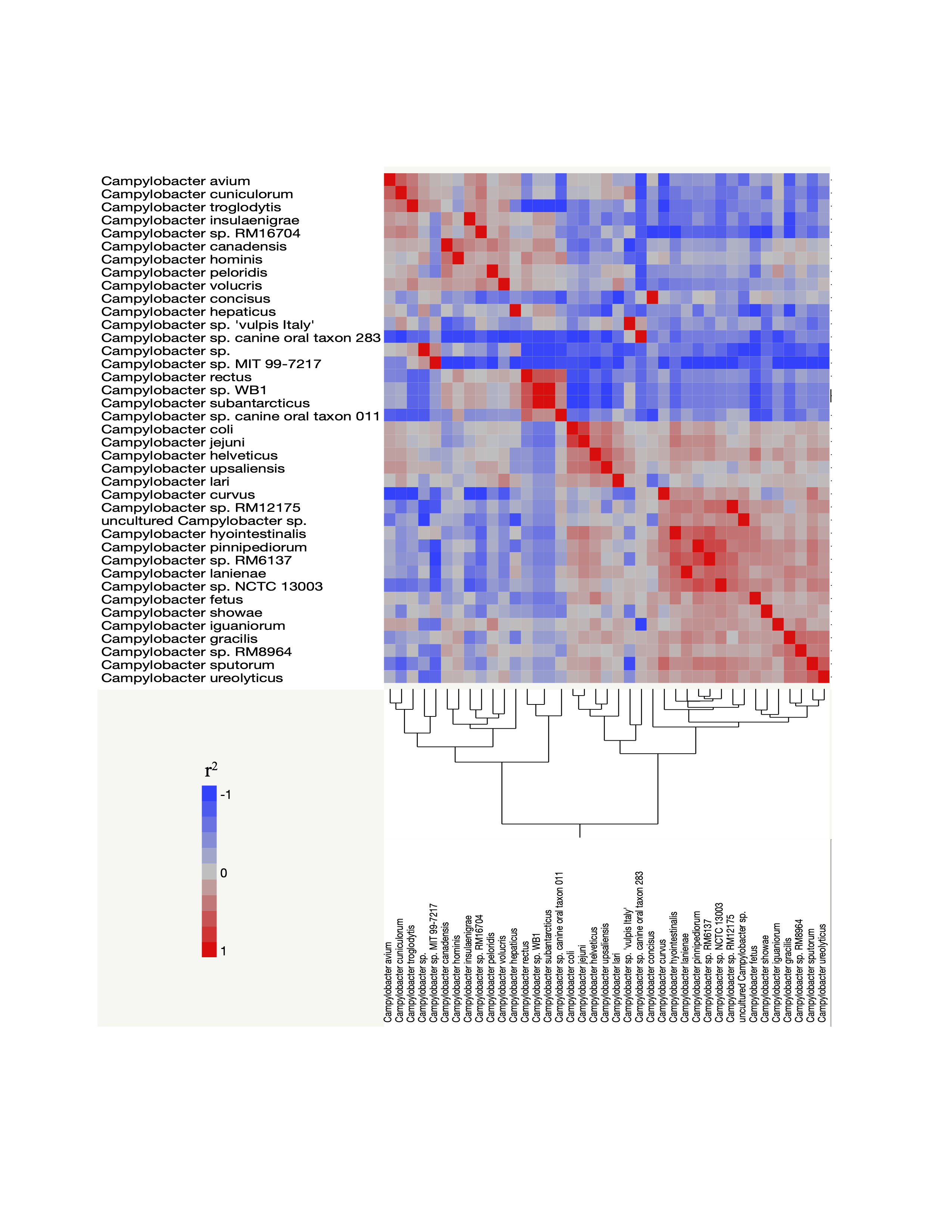

Supplement: Figure S3 — Co-occurrence of Campylobacter spp. in the children stool samples. Co-occurrence Heatmap profile was created using the multivariate analysis data (r2) based on the prevalence of Campylobacter spp. in the stool samples (see Figure 2). [file Image_3.JPEG]

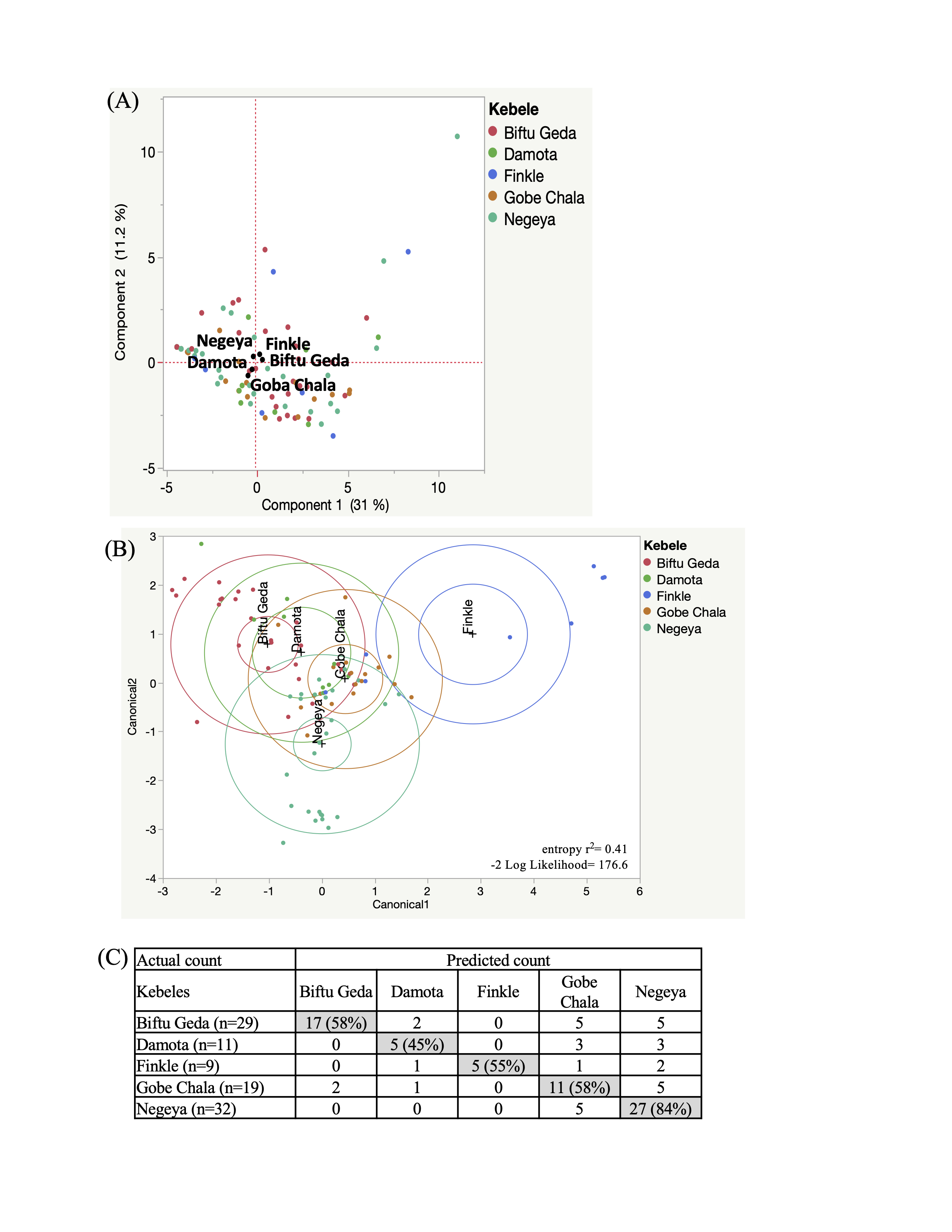

Supplement: Figure S4 — Campylobacter spp. profile between children stool samples based on the meta-total RNA sequencing (MeTRS). (A) Principal component analysis of the Campylobacter spp. diversity and abundance in the stools. Each colored dot represents one child stool sample (n = 100). Black dot represents the average profile for a given kebele (n = 5). (B) Discriminant analysis of the Campylobacter spp. diversity and abundance in the stools based on kebeles. The outside ellipse contains ~50% of the observations. The inside ellipse represents 95% confidence level (Cut-off for MeTRS data; contigs number ≥ 10; Read length ≥ 50; Z-score ≥ 1. (C). Clusterization profile of the stools based on the Campylobacter spp. diversity, prevalence, and abundance data. [file Image_4.JPEG]

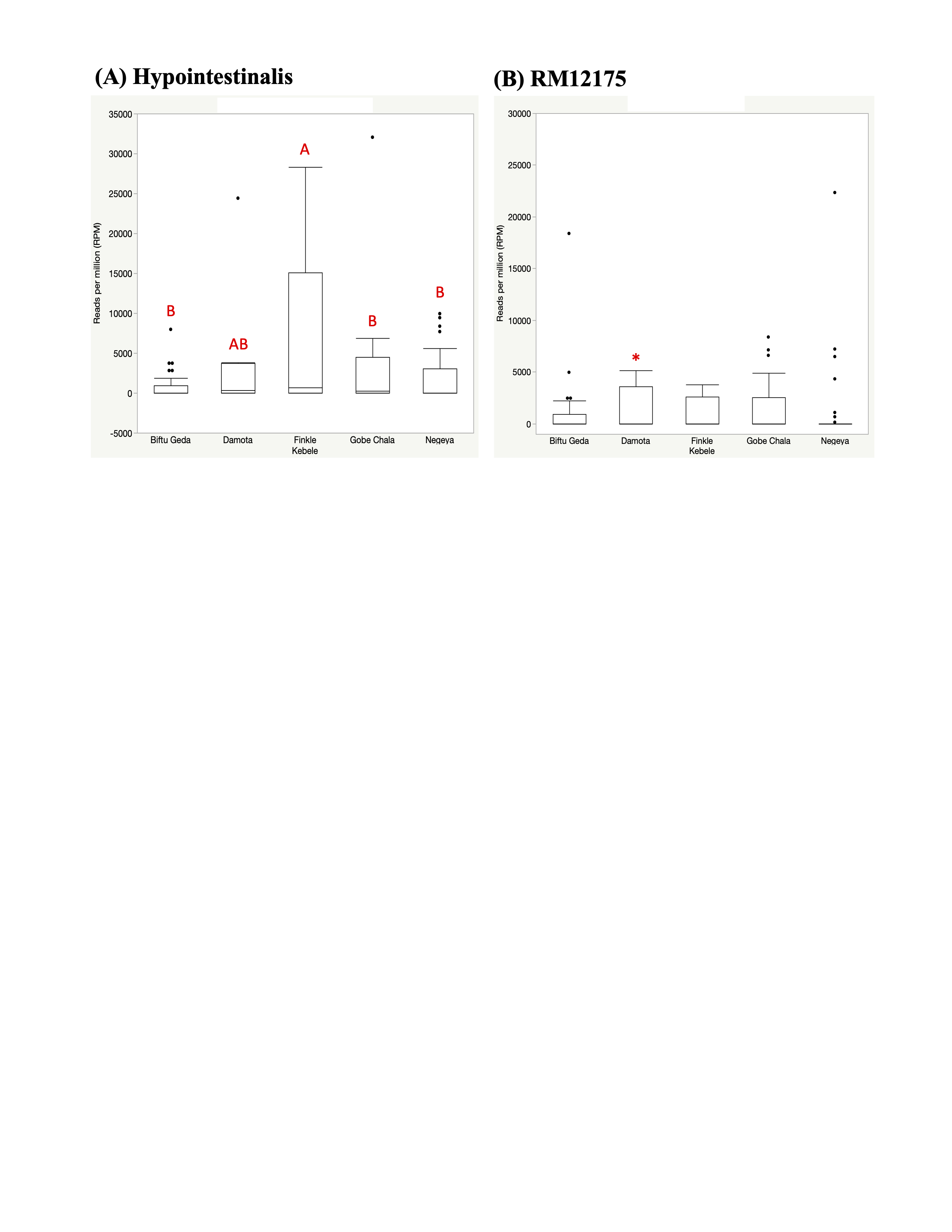

Supplement: Figure S5 — Differences in abundance of Campylobacter hyointestinalis and Campylobacter RM12175 between kebeles. Letters (A,B) indicates different statistical groups (P < 0.01). Star: read per million (rpm) are significantly higher in the designated kebele compared to the other kebeles. [file Image_5.JPEG]

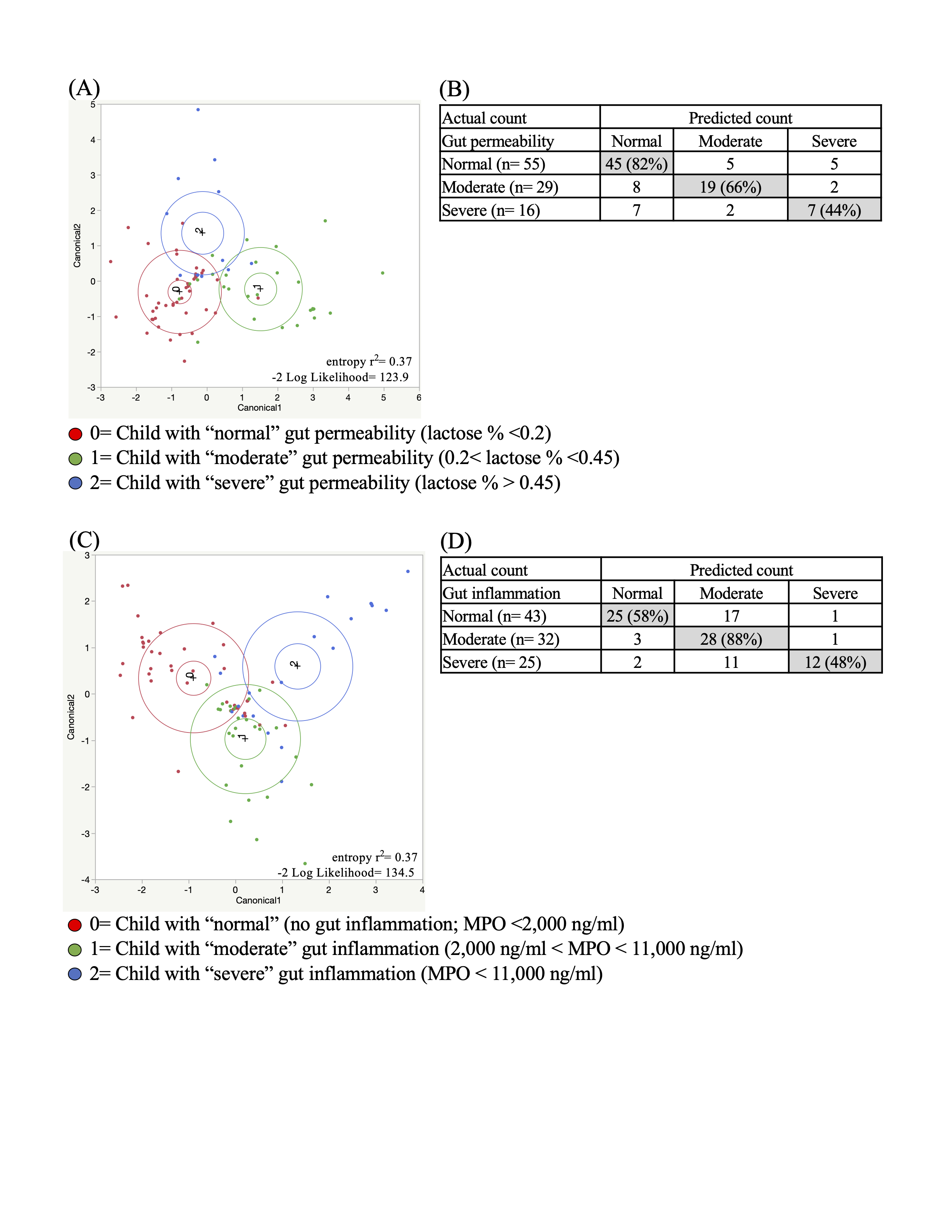

Supplement: Figure S6 — Campylobacter spp. profiles between children stool samples based EED severity and diarrhea. Discriminant analysis of the Campylobacter spp. abundance in the children stools based on (A) the gut permeability (lactulose%), (C) gut inflammation ([myeloperoxidase] in ng/ml), (E) EED severity, and (G) diarrhea prevalence data. The outside ellipse contains approximately 50% of the observations. The inside ellipse represents 95% confidence level. Clusterization profile of the Campylobacter spp. abundance in the stools based on (B) the gut permeability (lactulose%), (D) gut inflammation ([myeloperoxidase] in ng/ml), (F) EED severity, and (H) diarrhea prevalence data. Additional details concerning the EED severity determination are presented in Table S1. [file Image_6.JPEG]

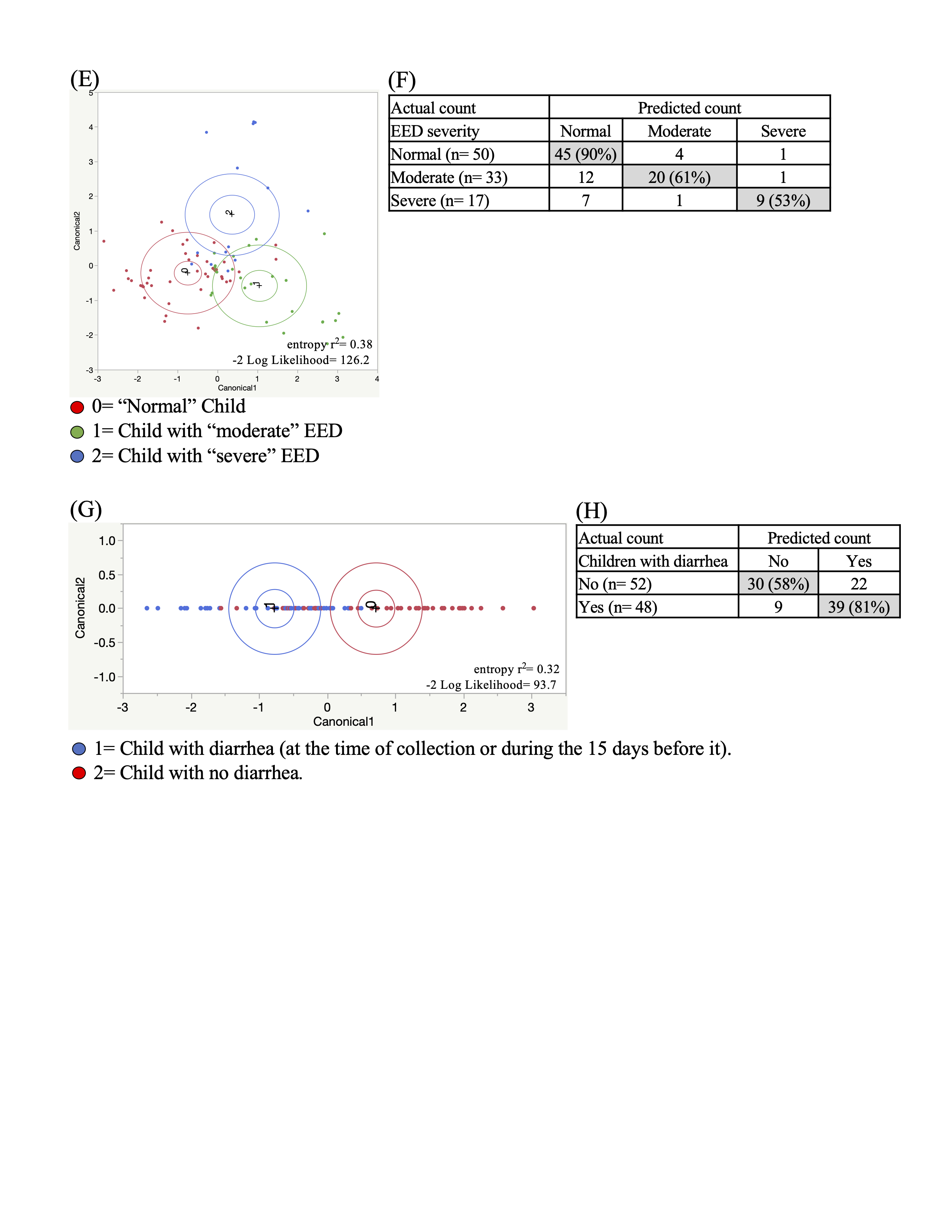

Supplement: Figure S7 — Microbiome profiles between children stool samples based on kebeles. (A) Principal component analysis of the children stool samples based on kebeles. Each colored dot represents one stool sample (n = 100). Kebeles with the same color code belong to the same cluster and therefore harbored equivalent microbiota diversity and abundance. A T2 test revealed a total of 11 outliers (stools with significantly different microbiota profile compared to the rest of the population; outside the red oval). (B) Discriminant analysis of the stool microbiome based on kebeles. The outside ellipse contains ~50% of the observations. The inside ellipse represents 95% confidence level. (C) Clusterization profile of the stool microbiome based on kebeles. [file Image_7.JPEG]

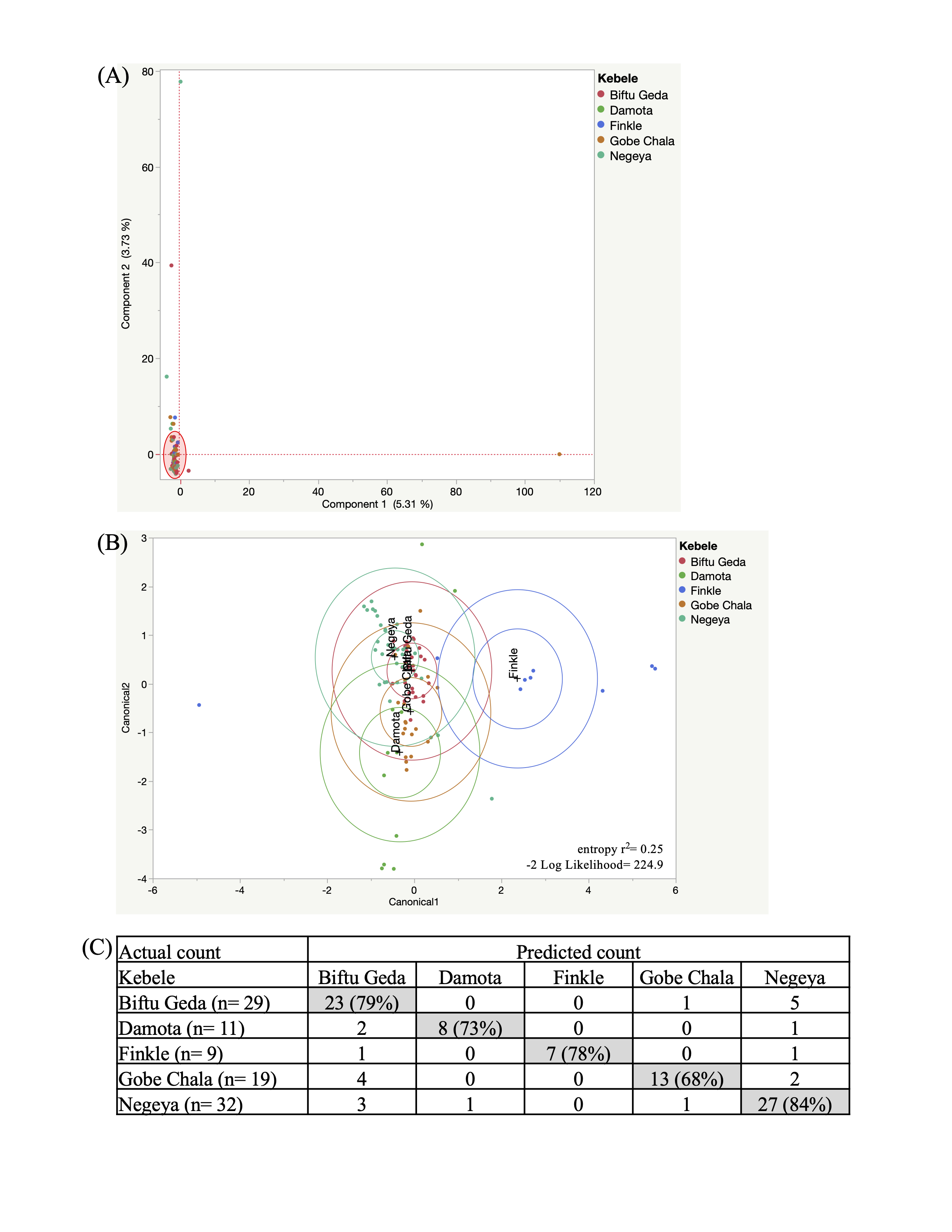

Supplement: Figure S8 — Microbiome profile based on Campylobacter prevalence. (A) Discriminant analysis of the children stool microbiome based on Campylobacter prevalence. The outside ellipse contains ~50% of the observations. The inside ellipse represents 95% confidence level. (B) Clusterization profile of the stool's microbiome based on Campylobacter prevalence. [file Image_8.JPEG]

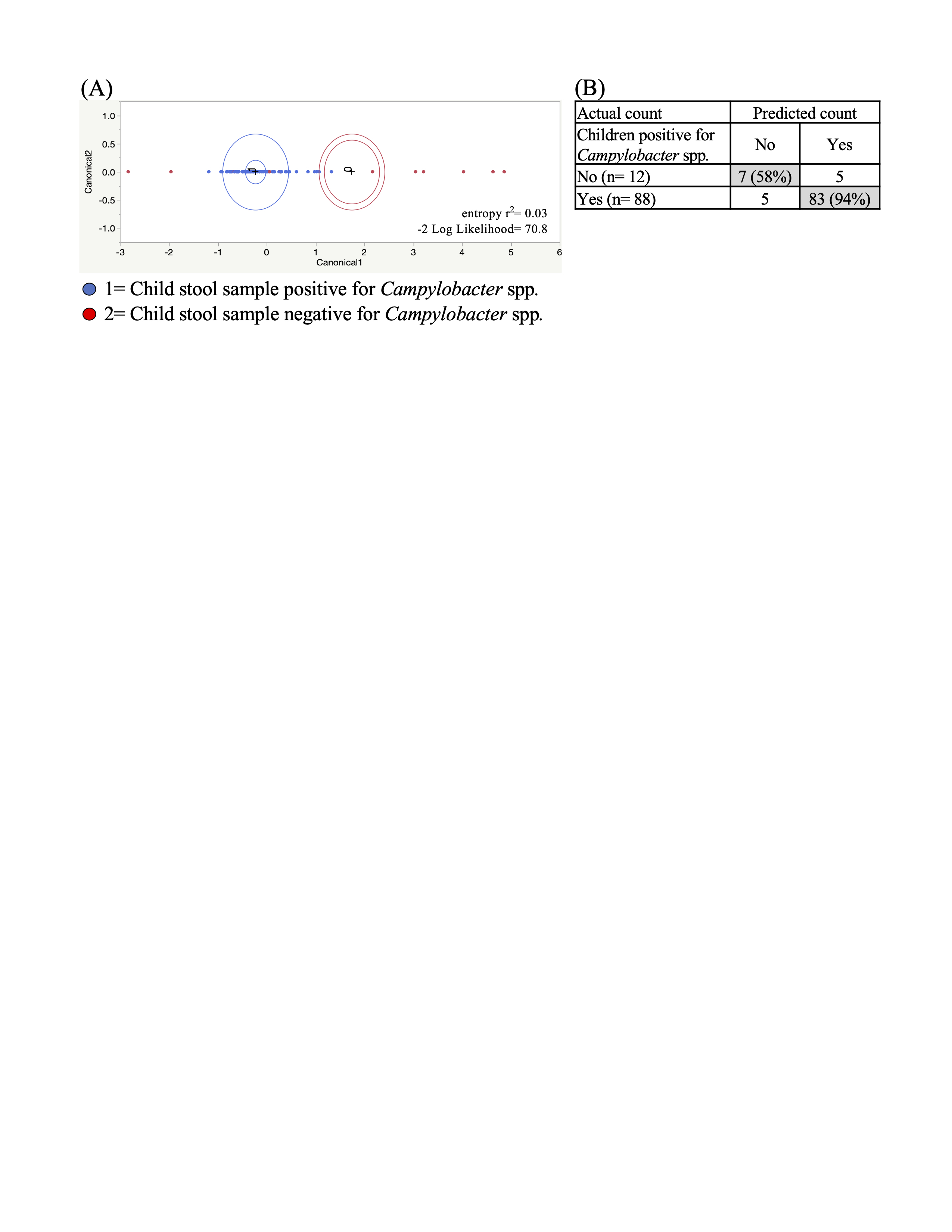

Supplement: Figure S9 — Microbiome profile based on environmental enteric dysfunction (EED). Discriminant analysis of the children stool microbiome composition based on (A) the gut permeability (lactulose%), (C) gut inflammation ([MPO] in ng/ml), (E) EED severity, and (G) diarrhea prevalence data. The outside ellipse contains ~50% of the observations. The inside ellipse represents 95% confidence level. Clusterization profile of the Campylobacter spp. abundance in the stools based on (B) the gut permeability (lactulose%), (D) gut inflammation ([MPO] in ng/ml), (F) EED severity, and (H) diarrhea prevalence data. Additional details concerning the EED severity determination are presented in Table S1. (I) Correlation between the specific bacterial species of the children stool microbiome and the gut permeability (lactulose%), gut inflammation ([MPO] in ng/ml), EED severity, and diarrhea prevalence data. N, number of stools positive for the designated bacterium. Values in the table represent the mean (log [read per million]) ± standard error for the designated bacterial species for a given status. Stars represent bacterial species harboring at least 2-fold difference in abundance between the “normal” and “severe” status for at least one of the parameters studied. MPO: myeloperoxidase. [file Image_9.JPEG]

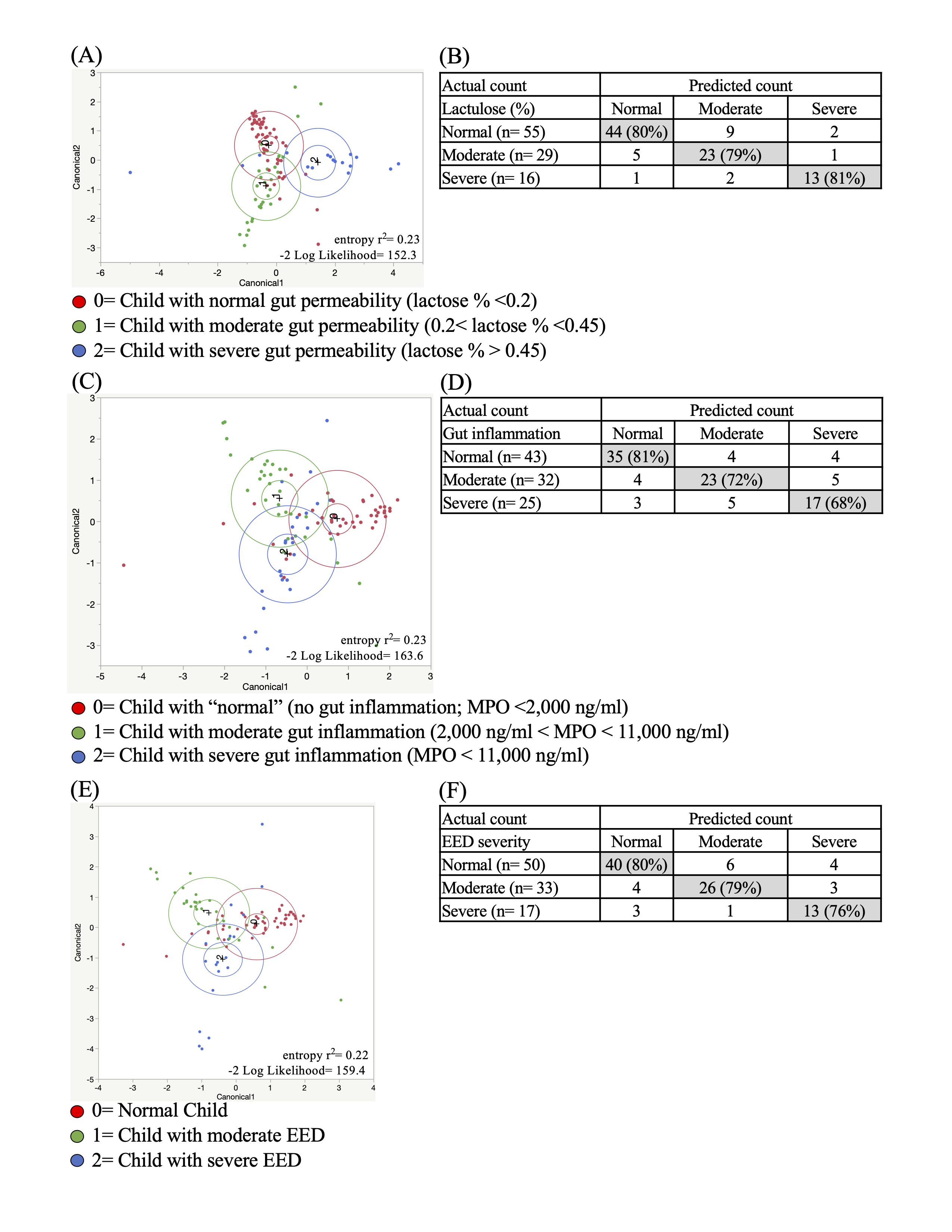

Supplement: Supplementary file 12 [file Image_10.JPEG]

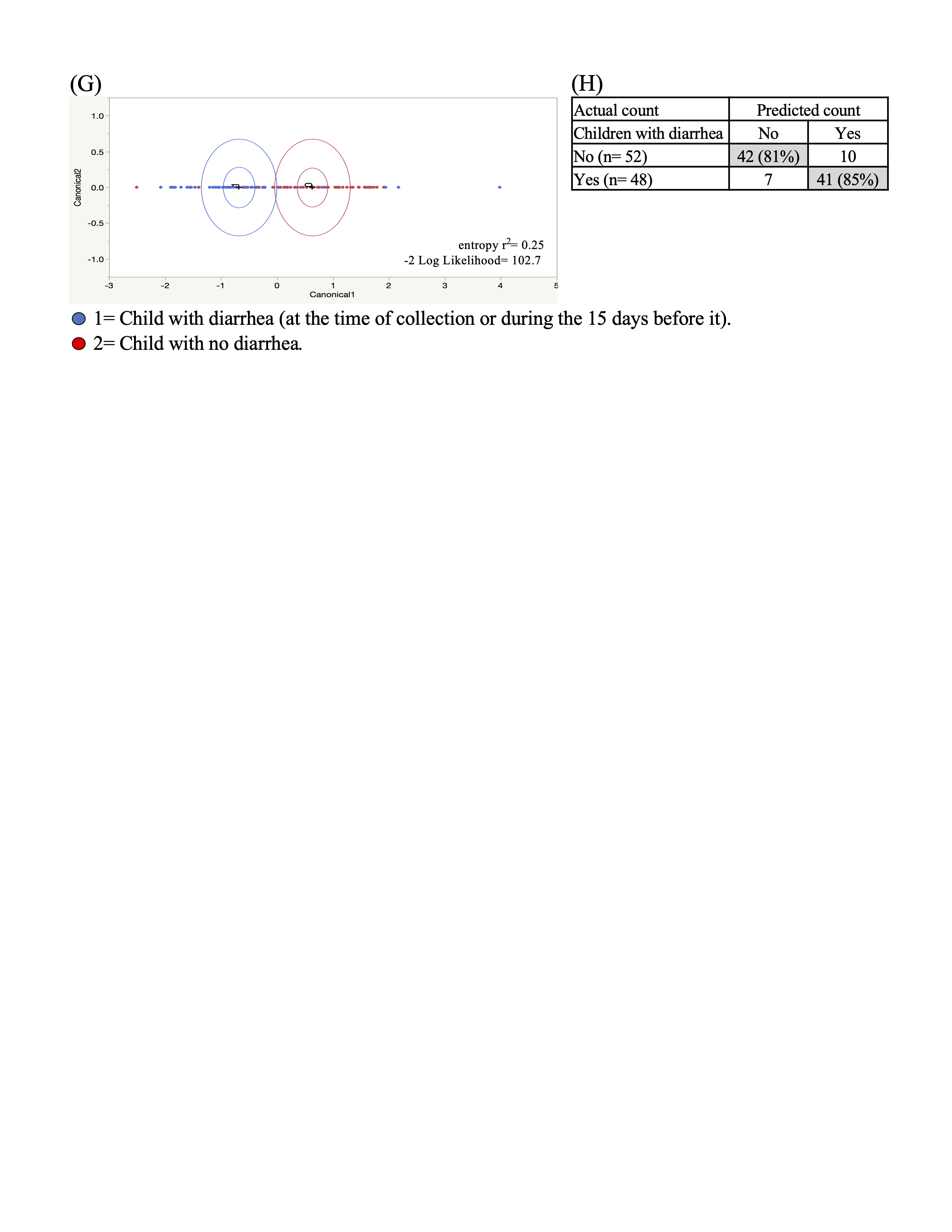

Supplement: Supplementary file 13 [file Image_11.JPEG]

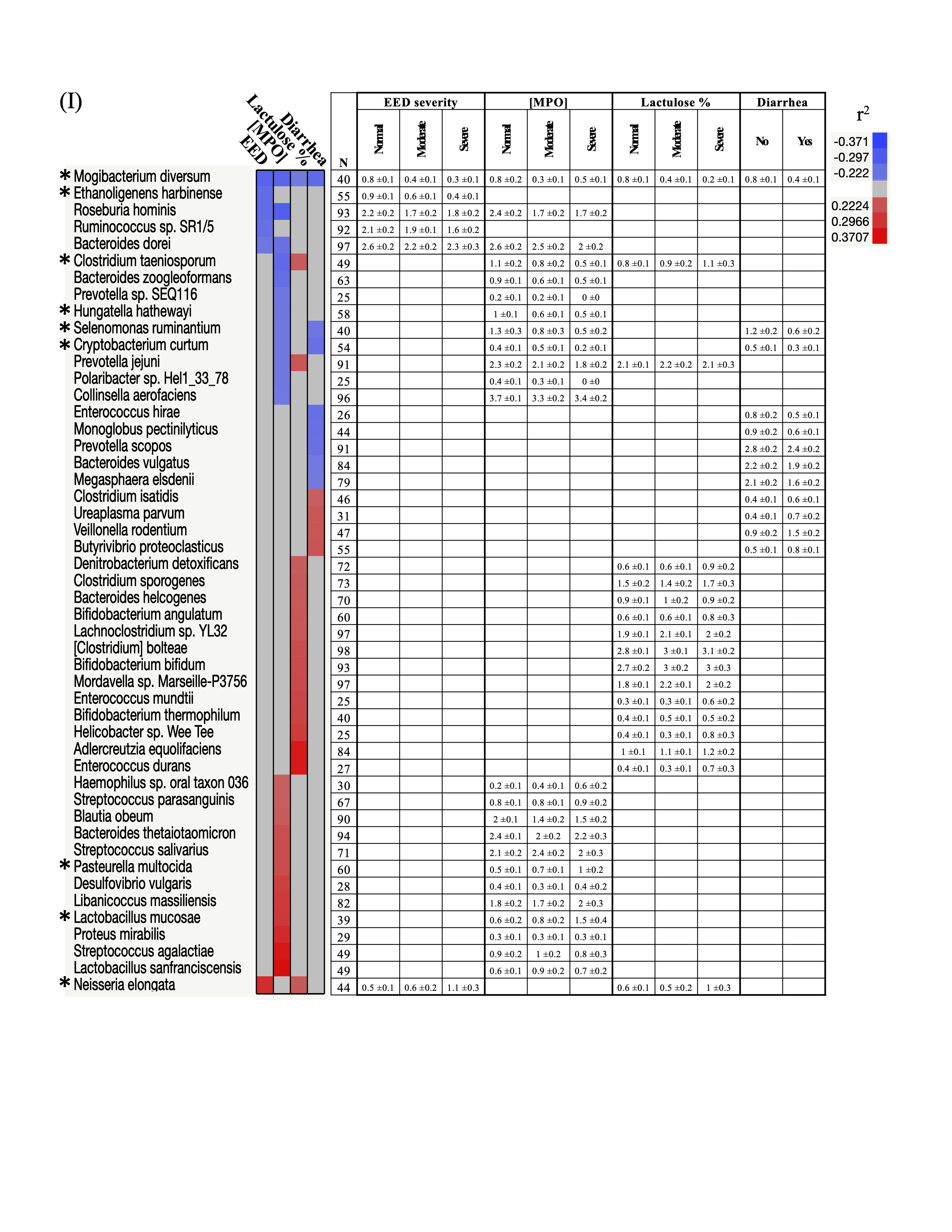

Supplement: Supplementary file 14 [file Image_12.JPEG]
