# Supplementary material for: Co-occurrence of Campylobacter Species in Children From Eastern Ethiopia, and Their Association With Environmental Enteric Dysfunction, Diarrhea, and Host Microbiome
Source: Front Public Health. 2020 Apr 15;8:99. doi: 10.3389/fpubh.2020.00099 (PMC7174729; doi:10.3389/fpubh.2020.00099)
Supplement: Table S1 — Primers used in the study. Source: Adapted from Deals et al. (28). [file Table_1.docx]

**Table S1 | Primers used in the study**

| **Target identification** | **Target gene** | **Primer sequence** | | **Product size (bp)** |
| --- | --- | --- | --- | --- |
|  |  | **Forward** | **Reversed** |  |
| ***Campylobacter* genus** | 16S RNA | ATCTAATGGCTTAACCATTAAAC | GGACGGTAACTAGTTTAG TATT | 857 |
| ***C. coli*** | *ceuE* | AATTGAAAATTGCTCCAACTATG | TGATTTTATTATTTGTAGCAGCG | 462 |
| ***C. jejuni*** | *mapA* | TATTTTATTTTTGAGTGCTTGTG | GCTTTATTTGCCATTTGTTTTATTA | 589 |

**Source: Adapted from Denis et al., 1999**
